# Direct neuronal reprogramming of mouse astrocytes is associated with multiscale epigenome remodeling and requires Yy1

**DOI:** 10.1038/s41593-024-01677-5

**Published:** 2024-07-02

**Authors:** Allwyn Pereira, Jeisimhan Diwakar, Giacomo Masserdotti, Sude Beşkardeş, Tatiana Simon, Younju So, Lucía Martín-Loarte, Franziska Bergemann, Lakshmy Vasan, Tamas Schauer, Anna Danese, Riccardo Bocchi, Maria Colomé-Tatché, Carol Schuurmans, Anna Philpott, Tobias Straub, Boyan Bonev, Magdalena Götz

**Affiliations:** 1 Biomedical Center Munich (BMC), Physiological Genomics, LMU Munich, Planegg, Germany; 2 Institute of Stem Cell Research, Helmholtz Center Munich, BMC LMU Munich, Planegg, Germany; 3 Helmholtz Pioneer Campus, Helmholtz Center Munich, Neuherberg, Germany; 4 Biological Science Platform, Sunnybrook Research Institute; Department of Biochemistry, University of Toronto, Toronto, ON Canada; 5 Biomedical Center Munich (BMC), Bioinformatic Core Facility, Faculty of Medicine, LMU Munich, Planegg, Germany; 6 Institute of Computational Biology, Helmholtz Center Munich, Neuherberg, Germany; 7 Biomedical Center Munich (BMC), Physiological Chemistry, Faculty of Medicine, LMU Munich, Planegg, Germany; 8 Wellcome–Medical Research Council Cambridge Stem Cell Institute, University of Cambridge, Cambridge Biomedical Campus, Cambridge, UK; 9 Excellence Cluster of Systems Neurology (SYNERGY), Munich, Germany; 10 Present Address: Nantes Université, CHU Nantes, INSERM, TaRGeT - Translational Research in Gene Therapy, UMR 1089, Nantes, France; 11 Present Address: Institute of Stem Cells and Epigenetics, Helmholtz Center Munich, Neuherberg, Germany; 12 Present Address: Department of Basic Neurosciences, University of Geneva, Geneva, Switzerland

**Keywords:** Molecular neuroscience, Reprogramming

## Abstract

Direct neuronal reprogramming is a promising approach to regenerate neurons from local glial cells. However, mechanisms of epigenome remodeling and co-factors facilitating this process are unclear. In this study, we combined single-cell multiomics with genome-wide profiling of three-dimensional nuclear architecture and DNA methylation in mouse astrocyte-to-neuron reprogramming mediated by Neurogenin2 (Ngn2) and its phosphorylation-resistant form (PmutNgn2), respectively. We show that Ngn2 drives multilayered chromatin remodeling at dynamic enhancer–gene interaction sites. PmutNgn2 leads to higher reprogramming efficiency and enhances epigenetic remodeling associated with neuronal maturation. However, the differences in binding sites or downstream gene activation cannot fully explain this effect. Instead, we identified Yy1, a transcriptional co-factor recruited by direct interaction with Ngn2 to its target sites. Upon deletion of Yy1, activation of neuronal enhancers, genes and ultimately reprogramming are impaired without affecting Ngn2 binding. Thus, our work highlights the key role of interactors of proneural factors in direct neuronal reprogramming.

## Abstract (web summary)

The molecular mechanisms underlying direct neuronal reprogramming are unclear. Here the authors show Ngn2-mediated chromatin remodeling and its binding sites underlying mouse astrocyte-to-neuron reprogramming and identify Yy1, a transcription co-factor, as an important regulator.

## Main

Direct neuronal reprogramming is a promising approach to replace neurons lost in disease^1^. Differentiated glial cells have been successfully reprogrammed into functional neurons by the forced expression of transcription factors (TFs), such as Neurogenin2 (Ngn2), achaete-scute homolog 1 (Ascl1), NeuroD1/4 and Pax6 in vitro^2–4^ and in vivo after brain lesion^5,6^. Although the transcriptional changes in TF-mediated direct neuronal reprogramming have been examined^4,7–11^, the mechanisms underlying epigenetic rewiring remain largely unclear^6,7,12^. For example, incomplete rewiring of the three-dimensional (3D) genome^13^ could contribute to incomplete or failed cellular reprogramming^14^, and DNA methylation is often viewed as key in regulating cell fate^15^, even though functional evidence rather points to a role in cell maturation^16^. Thus, how epigenome dynamics are coordinated during neuronal reprogramming is an important, yet unexplored, topic. Single-cell and bulk multiomic methods^17–19^ are well suited to explore alterations of these key epigenetic layers during direct neuronal reprogramming.

During development and reprogramming, proneural TFs, such as Ngn2, engage in a complex interplay with co-factors, epigenetic regulators and enhancer elements to precisely regulate the expression of their target genes, thereby controlling cellular identity transitions^8,20,21^. Although such regulatory interactions can be restricted by the 3D genome architecture, TFs can rewire 3D genome contacts themselves to enable the formation of cell-type-specific enhancer–promoter (E–P) interactions during development^19,22,23^. However, still too little is known of such dynamics during direct neuronal reprogramming to understand key TF-driven epigenetic remodeling events and their impact on cell fate change.

To link epigenome remodeling with the outcome of mouse astrocyte-to-neuron reprogramming, we aimed to compare TFs with different reprogramming efficiencies. The neurogenic activity of Ngn2 is negatively impacted by phosphorylation at several residues, including at a conserved on–off switch in the loop-helix 2 region of the basic helix-loop-helix (bHLH) domain^24^ in serine/threonine residues adjacent to prolines (SP/TP sites)^25–27^, and a tyrosine residue regulates neuronal migration^28^. In the present study, we chose the mouse 9S-A phosphomutant form of Ngn2 (PmutNgn2), resistant to phosphorylation by proline-directed serine kinases^25^ and showing stronger neurogenic activity during development and, as recently shown, in human iPSC-derived glia-to-neuron reprogramming^29^. Our study revealed that improved reprogramming by PmutNgn2 is accompanied by more extensive epigenetic changes, and it identified a direct interactor, Yy1, which is recruited by Ngn2 and is crucial for its activity.

## Results

### PmutNgn2 accelerates astrocyte-to-neuron reprogramming

**Fig. 1:**
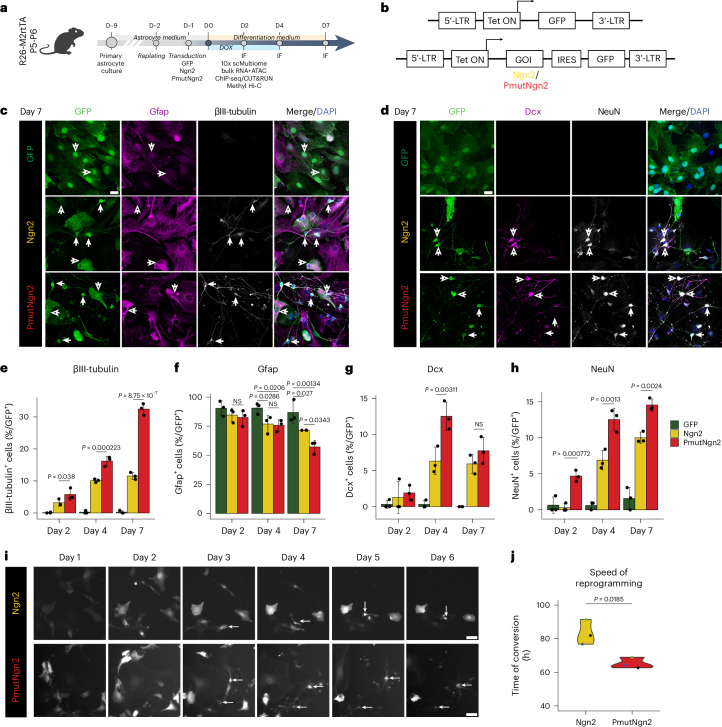
PmutNgn2 accelerates mouse astrocyte-to-neuron reprogramming. **a**,**b**, Scheme of the experimental design (**a**) and the lentiviral constructs (**b**) used in the study. **c**,**d**, Representative micrographs of astrocytes immunostained as indicated on top of the panels at 7 dpi for the experimental condition indicated on the left. Scale bar, 20 µm. In **c**, the filled arrows mark the iNs, and the empty arrows mark cells lacking neuronal markers (*n* = 3, biologically independent samples). In **d**, the filled arrow marks mature iNs (NeuN^+^Dcx^−^), and the empty arrow marks immature iNs (NeuN^+^Dcx^+^) (*n* = 3, biologically independent samples). **e**–**h**, Histograms showing percent of βIII-tubulin^+^ (**e**), Gfap^+^ (**f**), Dcx^+^ (**g**) and NeuN^+^ (**h**) cells among transduced cells (*y* axis) over time (*x* axis). Data are plotted as mean ± s.e.m.; each dot represents an individual biological replicate (*n* = 3). **i**, Micrographs show example frames of a continuous live-imaging experiment for the indicated experimental conditions across the indicated timescale. Scale bar, 50 µm. **j**, Violin plot showing the first timepoint when tracked cells showed a neuronal morphology; each dot represents a biological replicate (*n* = 3). Statistical significance was calculated using a linear regression model (**e**–**h**,**j**). D, day; NS, not significant; IF, immunofluorescence. Source data

**Extended Data Fig. 1:**
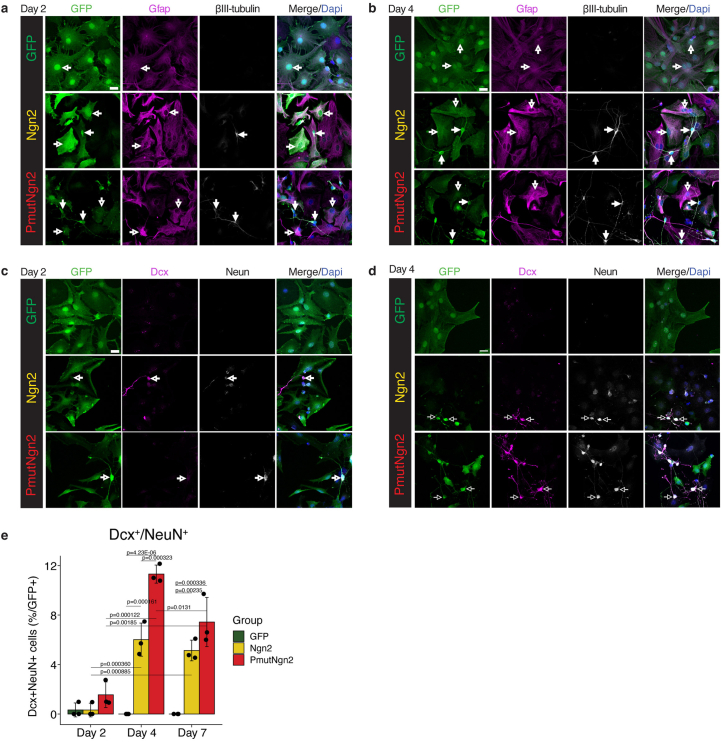
PmutNgn2 accelerates mouse astrocyte-to-neuron reprogramming. **(a-d)** Representative micrographs of astrocytes immunostained for the indicated proteins at day 2 and 4 after induction of the inducible constructs expressing GFP, Ngn2 or PmutNgn2. Scale bar 20 µm. In **(c)**, the filled arrow heads mark the iNs, the empty arrow heads mark example cells lacking neuronal markers. In **(d)**, the filled arrow heads mark mature iNs (NeuN+Dcx-), the empty arrow marks immature iNs (NeuN+Dcx + ). **(e)** Histograms depicting cell quantifications indicated on the y-axis over time indicated on the x-axis. Data are plotted as mean ± standard deviation (sd) where the dots represent individual biological replicates (n = 3).

Primary mouse cortical astrocytes from postnatal day (P) 5–6 mice were transduced with doxycycline (dox)-inducible lentiviral expression vectors^30^ coding for green fluorescent protein (GFP), Ngn2-IRES-GFP or PmutNgn2-IRES-GFP (Fig. 1a,b). Immunostaining for βIII-tubulin and Gfap to label neurons and astrocytes, respectively, at 2, 4 and 7 days post induction (dpi) showed a gradual increase in the proportion of induced neurons (iNs; for criteria, see ref. ^5^ and Methods) with a higher iN proportion and greater loss of Gfap achieved by PmutNgn2 compared to Ngn2 by 7 dpi (Fig. 1c–f and Extended Data Fig. 1a,b). PmutNgn2 iNs also exhibited more complex neurites (Fig. 1c), reminiscent of PmutAscl1-generated iNs^31^, and faster maturation, indicated by more neurons expressing the mature marker NeuN without the immature marker Doublecortin (Dcx) (Fig. 1d,g,h and Extended Data Fig. 1c–e). Continuous live-cell imaging^5,32^ confirmed that PmutNgn2-transduced cells acquired a neuronal-like morphology quicker (most cells converted by 64 h) than Ngn2-transduced cells (most converted by 84 h) (Fig. 1i,j and Supplementary Videos 1 and 2). Thus, PmutNgn2 instructs the formation of more neuronal cells compared to Ngn2, due, at least in part, to faster conversion and maturation.

### PmutNgn2 governs a neuron maturation transcriptional network

**Fig. 2:**
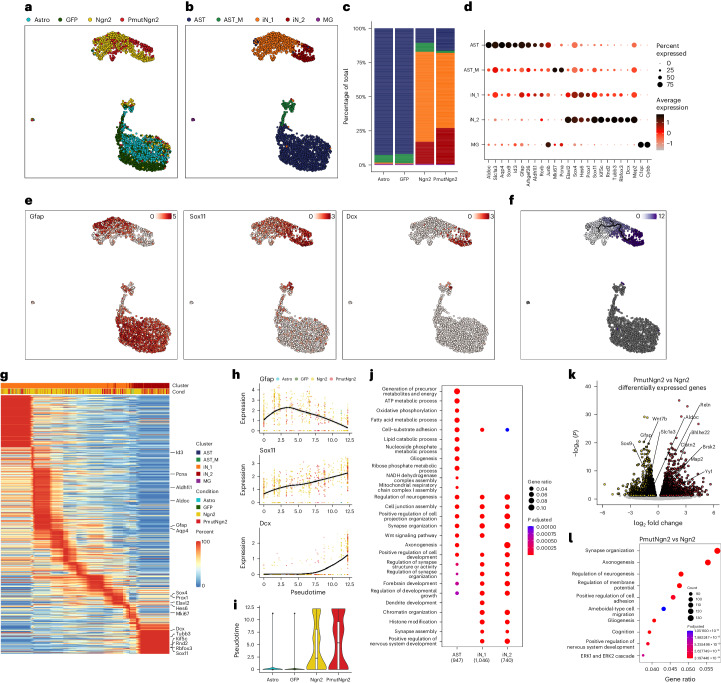
Gene expression differences elicited by Ngn2 and PmutNgn2. **a**,**b**, Joint (single-cell RNA + single-cell ATAC) UMAP projection, where murine cells are colored based on the experimental condition (**a**) or their cluster identity (**b**). **c**, Stacked bar plot representing the relative proportion of the identified cell types in each experimental condition. **d**, Dot plot showing the proportion of cells (as a percentage, size of dots) and the gene expression levels of selected marker genes (color) in the respective cell type clusters. **e**, UMAP visualization of the expression levels of indicated markers genes. **f**, UMAP visualization of the inferred neuronal maturation trajectory and the corresponding pseudotime. **g**, Heatmap depicting the expression levels of the most variable genes across maturation pseudotime. **h**, Gene expression changes of the indicated marker genes across the maturation pseudotime. Each dot depicts the expression level per cell of the given gene across pseudotime; the color indicates the experimental condition identity; and the line represents a smoothed fit of the gene expression values across pseudotime. **i**, Box plots depicting the pseudotime values per condition (*n* = 708, 708, 732 and 311 cells, respectively). Shown are the median (line), 25th or 75th percentiles (box) and 10th or 90th percentiles (whiskers). **j**, Bubble plot depicting the enriched GO terms specific to the indicated clusters. Statistical significance was calculated using a Benjamini–Hochberg-adjusted hypergeometric test. **k**, Volcano plot depicting the differentially expressed genes (FDR < 0.05) from a pairwise comparison between PmutNgn2 versus Ngn2 using bulk RNA-seq (*n* = 3 biological replicates). **l**, Bubble plot showing the top 10 GO terms of the differentially regulated genes from a pairwise comparison between PmutNgn2 versus Ngn2 bulk RNA-seq.

**Extended Data Fig. 2:**
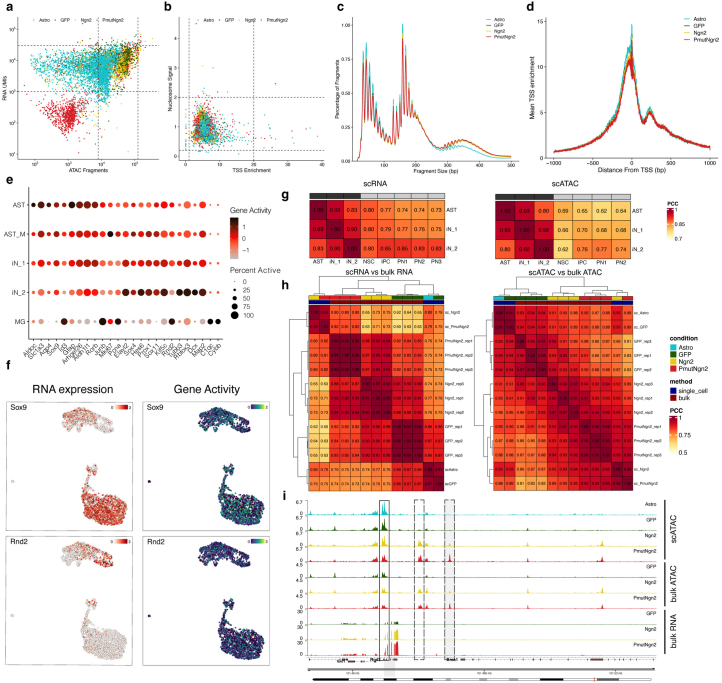
Quality control and comparison of genomic datasets. **(a-b)** Scatter plot of the RNA UMIs, ATAC fragments, Nucleosome signal and TSS enrichment per cell, colored by experimental condition. The cells within the square box were retained for further analysis. **(c)** Aggregated scATAC-seq (multiome) fragment size distribution, colored by cell condition. **(d)** Aggregated fragment enrichment at all TSSs from the scATAC-seq (multiome), colored by experimental condition. **(e)** Dot plot showing gene activity estimated by gene body accessibility at the indicated set of markers genes. **(f)** UMAP projection of RNA expression and gene activity for Sox9 and Rnd2 respectively. **(g)** Correlation heatmaps comparing this study with in vivo single-cell dynamics in the E14.5 developing mouse cortex^19^ **(h)** Correlation heatmaps showing the similarity between gene expression or ATAC using single cell (10x multiome) and bulk RNA/ATAC. **(i)** Genome track depicting the chromatin accessibility measured by single cell and bulk ATAC-seq and the transcriptional output measured by bulk RNA-seq at the *Rnd2* gene locus. Full rectangle demarcates the gene body of *Rnd2* while the dashed rectangles denote the putative distal regulatory regions of *Rnd2*.

To elucidate the enhanced reprogramming potency of PmutNgn2 compared to Ngn2, we simultaneously profiled the transcriptomic and chromatin accessibility landscape at single-cell resolution for untransduced astrocytes and astrocytes transduced with GFP only, Ngn2 or PmutNgn2 at 2 dpi using the 10x Genomics multiome platform (Supplementary Table 1). Integrated uniform manifold approximation and projection (UMAP) visualization^33^ revealed that Ngn2 and PmutNgn2 cells were distinct from both the untransduced and control astrocytes (Fig. 2a). Louvain clustering on the joint object gave five major clusters, including non-dividing astrocyte (AST) and dividing (mitotic) astrocyte (AST_M), neuronal populations iN_1 and iN_2 and a small cluster of microglial (MG) cells (Fig. 2b and Extended Data Fig. 2a–d). In line with phenotypic analysis, more PmutNgn2 cells were found in the mature iN_2 cluster (Fig. 2c), suggesting that PmutNgn2 induces a transcriptional program promoting neuronal maturation. Cells in both astrocyte clusters expressed astrocyte genes, including *Aldoc*, *Slc1a3* (*Glast*), *Aqp4*, *Sox9* and *Gfap* (Fig. 2d,e), with the added expression of *Mki67* and *Pcna* indicative of proliferation in the AST_M cluster (Fig. 2d). The pan-neurogenic Ngn2 target genes *Hes6*, *Prox1* and *Sox11* were expressed by iN_1 and iN_2, with iN_2 showing higher expression of neuronal genes—for example, *Dcx*, *Tubb3* and *Rbfox3*—than iN_1 (Fig. 2d,e). Chromatin accessibility at promoters and gene bodies (referred to as ‘gene activity’) did not always match transcriptional changes (Extended Data Fig. 2b–f), consistent with previous reports^19^, thus highlighting the importance of simultaneous profiling of both modalities in single cells.

Pseudotime analysis^34–37^ indicated a gradual transition from an immature neuronal state (iN_1) toward a relatively more mature neuronal state (iN_2) (Fig. 2f), characterized by the downregulation of astrocyte genes (for example, *Gfap*) and the increased expression of neuronal genes (for example, *Dcx*, *Tubb3* and *Rbfox3*), including TFs (for example, *Sox11*) (Fig. 2g,h). Also, genes regulating migration, for example the direct Ngn2 target *Rnd2* (ref. ^38^), were upregulated in iN_2, which displayed the highest correlation with intermediate progenitor cells and early post-mitotic neurons from the developing embryonic day (E) 14 mouse neocortex^19^ (Extended Data Fig. 2g). Furthermore, PmutNgn2 iNs had higher pseudotime values, suggesting further progression along the maturation trajectory (Fig. 2f–i). Gene Ontology (GO) term enrichment analysis revealed characteristic astrocyte functions, such as ‘fatty acid metabolic process’ and ‘lipid catabolic process’^32,39^ in the AST cluster and ‘synapse assembly’ or ‘positive regulation of nervous system development’ in the iN_1 and iN_2 clusters (Fig. 2j).

To further scrutinize differences between Ngn2 and PmutNgn2 iNs, we generated bulk RNA sequencing (RNA-seq) and assay for transposase-accessible chromatin using sequencing (ATAC-seq) data at the same timepoint (2 dpi), which were highly correlated with the single-cell data (Extended Data Fig. 2h). Pairwise comparison highlighted the higher expression of astrocytic genes (for example, *Sox9*, *Gfap* and *Aldoc*) in Ngn2 iNs and the upregulation of neuronal maturation genes (for example, *Reln* and *Brsk2*) in PmutNgn2 iNs (Fig. 2k). Consistent with the GO analysis (Fig. 2j), neurogenic TFs—for example *Bhlhe22* (ref. ^40^)—and chromatin regulators—for example, *Yy1* (ref. ^41^)—were upregulated upon PmutNgn2 expression (Fig. 2k) alongside GO terms associated with neuronal maturation (Fig. 2l). Overall, the single-cell multiome and bulk RNA-seq data demonstrated that PmutNgn2 accelerates neuronal conversion and maturation.

### Ngn2 remodels enhancer–gene interaction sites

Next, we examined the chromatin accessibility in our single-cell multiome dataset. Both genome-wide comparison and visualization at individual loci revealed strong correlation between single-cell and bulk ATAC-seq (Extended Data Fig. 2h,i). For instance, bulk and single-cell ATAC showed significant accessibility at a known Ngn2-regulated enhancer in the *Rnd2* locus after Ngn2 or PmutNgn2 expression^38^, whereas another putative enhancer became accessible only upon PmutNgn2 expression (Extended Data Fig. 2i).

**Fig. 3:**
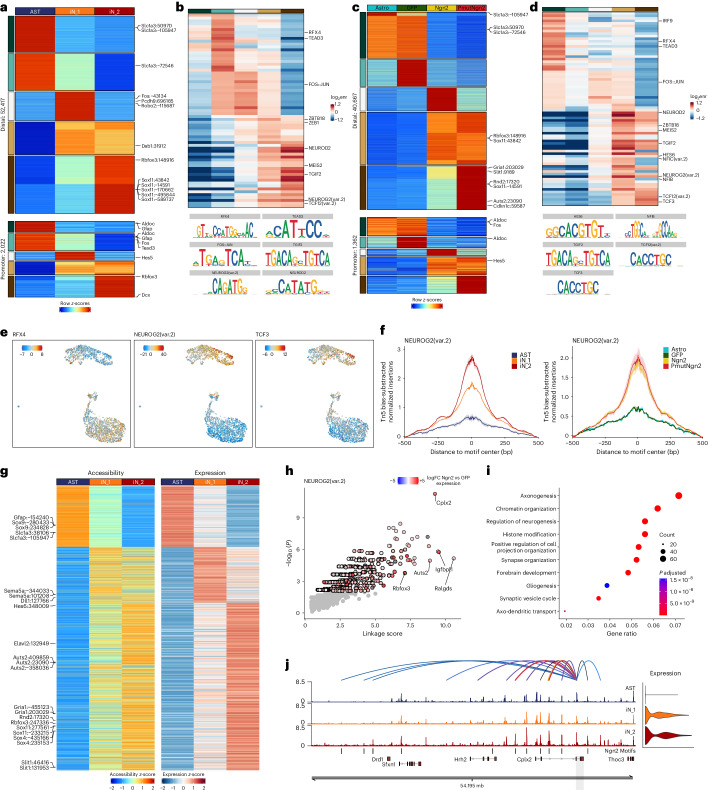
Changes in regulatory landscape caused by Ngn2 or PmutNgn2. **a**, Heatmap of the pseudobulk accessibility (*z*-scores) per murine cell type cluster of the differentially accessible sites in distal and promoter regions. **b**, Heatmap depicting the log_2_ fold enrichment of TF motifs in the five clusters identified by *k*-means clustering of the differentially accessible sites between cell types in distal and promoter regions. **c**, Same as **a** but for differentially accessible sites identified per experimental condition. **d**, Same as **b** but for TF motif enrichment in the clusters of differentially accessible sites identified per experimental condition. **e**, UMAP projection of motif activity scores for the indicated TFs. Scale bar represents the ChromVar motif deviation score. **f**, Motif footprint of NEUROG2(var.2) normalized for Tn5 insertion bias at the single-cell ATAC peaks in the indicated cell type clusters or experimental condition. **g**, Heatmaps depicting the aggregated accessibility of putative enhancer elements (left) and the expression levels of their linked genes (right) for positively correlated EGPs. **h**, Scatter plot depicting the predicted target genes of Ngn2 based on its motifs, with each dot representing a putative target gene. Significant genes are colored based on log fold change (FC) of expression between Ngn2 and the GFP condition (bulk RNA-seq). **i**, Bubble plot depicting the top 10 GO terms of the predicted Ngn2 target genes based on its motif. Color and size of circles indicate Benjamini–Hochberg-adjusted *P* value (hypergeometric test) and number of genes, respectively. **j**, Genome track showing aggregated single-cell ATAC accessibility and expression in the indicated experimental conditions at the *Cplx2* locus. Values represent reads per million mapped reads (RPM). enr, enrichment.

**Extended Data Fig. 3:**
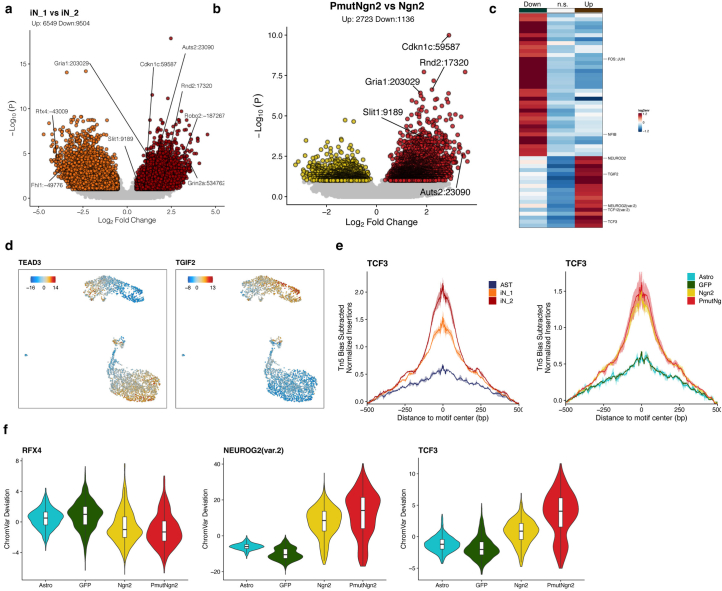
Chromatin accessibility characteristics of induced neurons. **(a-b)** Volcano plot depicting the differentially accessible regions (DARs) (pseudobulk chromatin accessibility at ATAC peaks from multiome data) identified from a pairwise comparisons. **(c)** Heatmap depicting the log2-fold enrichment of TF motifs in the DARs. **(d)** UMAP visualization of TF motif activity of the indicated TFs. **(e)** Motif footprint of Tcf3 normalized for Tn5 insertion bias at the scATAC peaks in the indicated cell type clusters (left) or experimental condition (right). **(f)** Boxplots depicting the ChromVar deviation values for the indicated TFs in the indicated experimental conditions (n = 708, 708, 732 and 311 cells per condition respectively). Boxplots display median (line), 25th or 75th percentiles (box) as well as 10th or 90th percentiles (whisker).

Next, we identified 52,417 promoter-distal and 2,022 promoter-proximal differentially accessible regions based on the single-cell multiome data, which clustered into five groups (*k*-means clustering, *k* = 5) (Fig. 3a). Regions associated with astrocyte genes, such as *Slc1a3* and *Aldoc*, showed highest accessibility in the AST cluster, whereas those associated with neuronal genes, including *Dcx* and *Rbfox3*, were more accessible in the iN clusters (Fig. 3a and Extended Data Fig. 3a). TF motif analysis of distal elements in each of these clusters revealed astrocyte TFs (Tead3 and Rfx4)^42^ enriched in clusters 1 and 2 (Fig. 3b). Conversely, clusters 4 and 5, more accessible in iNs, contained motifs of known neurogenic regulators, such as NeuroD2, Meis2 and Tcf12, as well as others previously not described, for example Tgif2 (Fig. 3b).

Using pseudobulk aggregation of the single-cell data, we compared chromatin accessibility dynamics and enrichment of associated TF motifs (for example, Rfx4 and Ap-1; Fos:Jun) per experimental condition (Fig. 3c,d). Astrocyte genes had the highest accessibility in untransduced or control astrocytes (Fig. 3c,d). Distal regions uniquely accessible upon PmutNgn2 induction were enriched for other E-box protein motifs, for example Tcf3 and Tcf12 (Fig. 3c,d), which can form heterodimers with Ngn2 to bind and transactivate target genes^43^. Pairwise comparison and motif enrichment analysis further confirmed these findings (Extended Data Fig. 3b,c). Motifs enriched in the AST cluster—for example, Rfx4 and Tead3—became closed, whereas motifs associated with Ngn2, Tcf3 and Tgif2 acquired accessibility during reprogramming (Fig. 3e,f and Extended Data Fig. 3d–f). Notably, Ngn2 and Tcf3 motifs were most accessible in the iN_2 cluster and in the PmutNgn2 condition (Fig. 3e–f and Extended Data Fig. 3d–f), suggesting that neuronal maturation is associated with increased chromatin remodeling at these regions.

To associate chromatin accessibility dynamics with gene expression changes^19,44^, we identified 7,917 positively correlated enhancer–gene pairs (EGPs) (Fig. 3g). These EGPs include known direct targets of Ngn2 (for example, *Rbfox3* and *Cplx2*)^45^ as well as predicted targets of Ngn2 previously not described (Fig. 3h). GO analysis revealed ‘regulation of neurogenesis’, ‘axonogenesis’ and ‘synapse organization’ among the top 10 terms, consistent with Ngn2 promoting neuronal fate (Fig. 3i). For example, the distal regulatory elements at the *Cplx2* locus (a synapse-associated protein-coding gene^46^) contained multiple Ngn2 motifs and became highly accessible in iN_2, which correlated with the upregulation of *Cplx2* in reprogramming (Fig. 3j).

These data indicate that Ngn2 drives widespread chromatin remodeling that is further enhanced by PmutNgn2. Thus, accelerated neuronal reprogramming elicited by PmutNgn2 is reflected by broader epigenome reorganization.

### Ngn2 chromatin binding activates neuronal enhancers

**Fig. 4:**
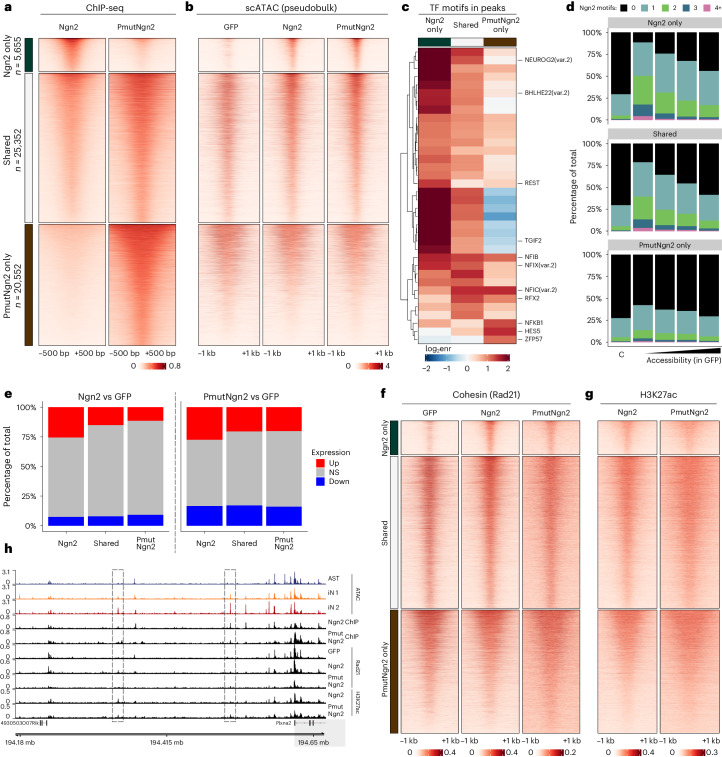
Ngn2 or PmutNgn2 differentially bind and remodel chromatin. **a**,**b**, Heatmaps showing the enrichment of ChIP-seq (**a**) or pseudobulk single-cell ATAC-seq (**b**) signal around differentially bound or shared peaks in murine iNs. **c**, Heatmaps depicting the motif enrichment in the peaks groups shown in **a** and **b**. **d**, Number of Ngn2 motifs in different peak categories, stratified by chromatin accessibility in GFP-transduced astrocytes. ‘C’ indicates the control group of peaks (randomly sampled accessible regions that are not bound by Ngn2 or PmutNgn2). **e**, Percentage of differentially regulated genes (based on bulk RNA-seq) overlapping with different peak categories (±100-kb window around the TSS). **f**,**g**, Heatmaps showing the enrichment of cohesin (Rad21) (**f**) or H3K27ac (**g**) CUT&RUN signal around differentially bound or shared Ngn2/PmutNgn2 peaks. **h**, Genome track showing aggregated single-cell ATAC accessibility, Ngn2/PmutNgn2 ChIP-seq as well as Rad21 and H3K27ac CUT&RUN tracks in the indicated experimental conditions at the *Plxna2* locus. Values represent reads per million mapped reads (RPM). Dashed rectangles depict distal enhancers of the *Plxna2* locus (shaded region), which show increased chromatin accessibility and recruitment of Rad21 and H3K27ac upon Ngn2 binding. enr, enrichment; NS, not significant.

**Extended Data Fig. 4:**
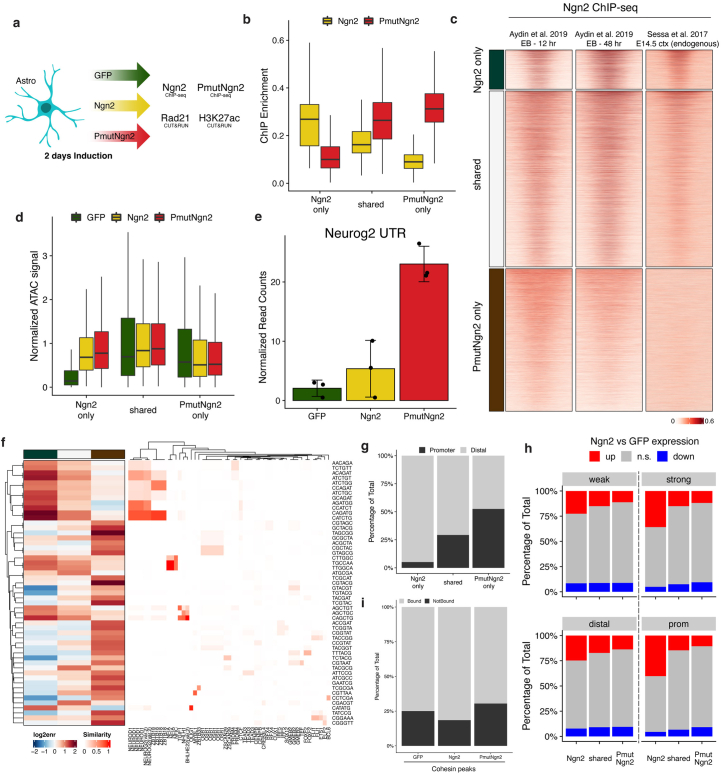
Ngn2 and PmutNgn2 binding remodels chromatin. **(a)** Schematic representation of the experimental setup **(b)** Boxplots depicting the ChIP-seq enrichment in reads per million (RPM) at the indicated peak groups (n = 5655, 25352 and 20552 regions respectively). **(c)** Heatmaps showing ChIP-seq around differentially bound or shared Ngn2/PmutNgn2 peaks. **(d)** Boxplots depicting normalized accessibility (RPM) at the indicated peak groups (n = 5655, 25352 and 20552 regions respectively). **(e)** Barplots with mean ± s.d. showing the normalized read counts mapping to either the 5’ or the 3’UTR of the endogenous Ngn2 locus. Dots represent individual biological replicates (n = 3) **(f)** Heatmaps depicting kmer enrichment in the peak groups shown in Fig. 4a,b, as well as the closest matching TF motif based on similarity. **(g)** Percentage overlap between peaks and gene promoters ( ± 5 kb from TSS). **(h)** Percentage of differentially regulated genes (based on bulk RNA-seq) overlapping with different peak categories or genomic features. **(i)** Percentage of Ngn2/PmutNgn2 peaks overlapping with Rad21 (Cohesin) peaks in the corresponding condition. All boxplots display median (line), 25th or 75th percentiles (box) as well as 10th or 90th percentiles (whisker).

To characterize Ngn2 and PmutNgn2 binding, we performed chromatin immunoprecipitation sequencing (ChIP-seq) for both conditions (Extended Data Fig. 4a) and identified 5,655 Ngn2-specific, 20,552 PmutNgn2-specific and 25,352 shared peaks (Fig. 4a), congruent with previous work showing increased binding of PmutAscl1 (ref. ^47^). Moreover, PmutNgn2 binding was stronger on the shared peaks (Extended Data Fig. 4b). Most of the Ngn2-specific and shared peaks were also occupied by endogenous Ngn2 (ref. ^48^) in the developing mouse cortex or upon Ngn2 induction in embryoid bodies^20^ (Extended Data Fig. 4c). Interestingly, PmutNgn2-specific sites were also bound by Ngn2 in embryonic bodies, suggesting that a fraction of Ngn2 is not phosphorylated in this system, whereas Ngn2 binding is not observed at the PmutNgn2-specific sites in E14 cortex, where Ngn2 is increasingly phosphorylated^26^.

Examining chromatin accessibility, Ngn2-specific peaks were opened upon reprogramming, consistent with the role of Ngn2 as a pioneer TF (Fig. 4b and Extended Data Fig. 4d). Conversely, most PmutNgn2-specific peaks were already accessible in astrocytes and remained accessible during conversion (Fig. 4b). Shared peaks were also already lowly accessible in astrocytes and increased in accessibility upon PmutNgn2 expression. Interestingly, expression analysis of the 5′ and 3′ untranslated regions (UTRs) at the endogenous *Ngn2* in the bulk RNA-seq data showed its increase upon PmutNgn2 expression, indicating that the increased accessibility at the Ngn2-specific peaks in the PmutNgn2 condition might be due to the upregulation of endogenous Ngn2 (Extended Data Fig. 4e).

To understand the differential binding pattern of Ngn2 and PmutNgn2, we asked which TF-binding motifs were enriched in each peak group. Ngn2-specific or shared peaks were enriched for the Ngn2 motif itself, whereas very few if any specific motif variants were enriched in the PmutNgn2-specific regions (Fig. 4c and Extended Data Fig. 4f). We further explored if the presence of multiple Ngn2 motifs in a peak influenced binding in relation to chromatin accessibility in GFP-transduced astrocytes: at both Ngn2-specific and shared peaks, the presence of multiple motifs correlated with increased binding at low accessible regions, suggesting a synergistic effect (Fig. 4d). Conversely, PmutNgn2-specific binding sites were mostly in promoter regions, where no such relationship between motif number and binding was observed (Fig. 4d and Extended Data Fig. 4g).

To explore the consequences of Ngn2 binding on transcription, we compared the percentage of differentially regulated genes (from bulk RNA-seq) that overlapped with different peak categories. Ngn2/PmutNgn2 binding preferentially led to gene activation (Fig. 4e). This effect was most prominent at Ngn2-specific peaks, where stronger binding at promoters resulted in higher number of upregulated genes (Extended Data Fig. 4h). Like the changes in chromatin accessibility, we also observed the upregulation of Ngn2-specific genes in the PmutNgn2 condition, potentially due to endogenous Ngn2 (Fig. 4e and Extended Data Fig. 4h).

Next, we asked if the increased accessibility at Ngn2 sites is also accompanied with enhancer activation. Both Rad21 (subunit of the cohesin complex) and H3K27ac (a histone modification associated with active enhancers) were present at these regions (as well as at shared and PmutNgn2 peaks), suggesting that Ngn2 binding results in chromatin remodeling, indicative of enhancer activation (Fig. 4f,g and Extended Data Fig. 4i). This is exemplified at the *Plnxa2* locus, a semaphorin co-receptor, where Ngn2 binding at distal enhancers is accompanied by increased chromatin accessibility, recruitment of Rad21 and increased H3K27ac (Fig. 4h).

These results suggest that Ngn2 and PmutNgn2 bind to overlapping, but also distinct, regions within the genome. Although Ngn2 binds strictly to its motif even at low accessible sites, PmutNgn2 preferentially binds promoters and already accessible regions. Both Ngn2 variants induce chromatin remodeling and activation of gene expression, for example by the recruitment of cohesin and deposition of H3K27ac.

### PmutNgn2 enhances chromatin rewiring and DNA demethylation

**Fig. 5:**
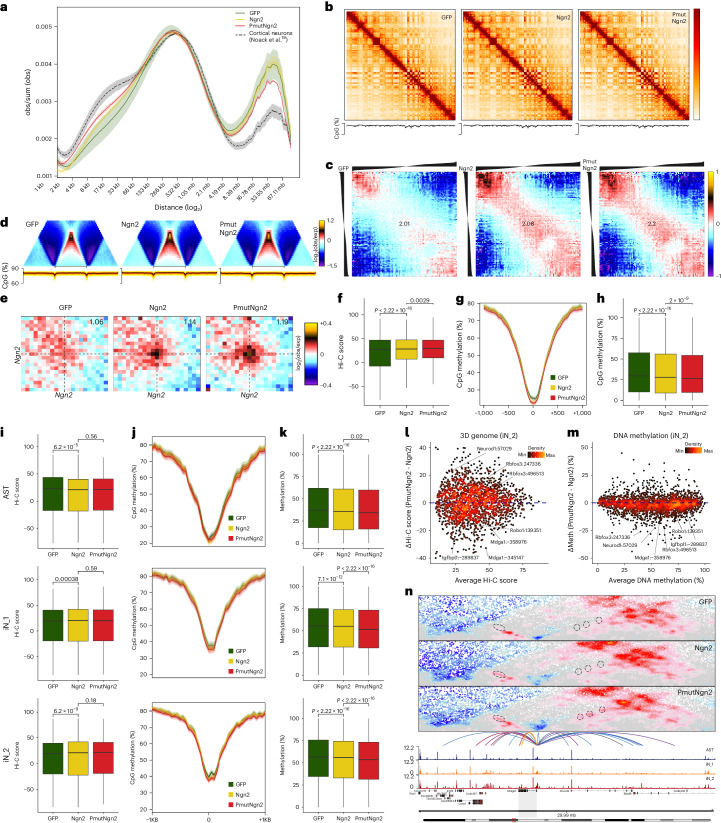
PmutNgn2 enhances 3D genome and DNA methylation changes. **a**, Contact probability as a function of the genomic distance. Lines: mean values from biological replicates; semi-transparent ribbons: s.e.m. obs/sum (obs), normalized contact probability. **b**, Knight–Ruiz balanced contact matrices for Chr 3 at 250-kb resolution (top) and DNA methylation (bottom). **c**, Average contact enrichment between pairs of 250-kb loci arranged by their eigenvalue (shown on top). Numbers represent the compartment strength. **d**, Average contact enrichment (top) and DNA methylation levels (bottom) across TADs. **e**, Aggregated Hi-C plots between intra-TAD pairs of the top 5,000 Ngn2 ChIP-seq peaks. **f**, Quantification of the interaction strength of intra-TAD contact pairs depicted in **e** (*n* = 6,888 pairs). **g**, Average DNA methylation plots at the distal regions within the top 5,000 Ngn2 ChIP-seq peaks. **h**, Quantification of the average DNA methylation at the sites depicted in **g** (*n* = 4,125 regions). **i**, Box plots depicting the interaction strength of intra-TAD cluster-specific positively correlated EGPs (*n* = 1,853, 1,795 and 3,852 pairs, respectively). **j**, Average DNA methylation plots at enhancers belonging to cluster-specific EGPs. **k**, Quantification of DNA methylation levels at enhancers belonging to cluster-specific EGPs (*n* = 1,568, 1,661 and 3,275 regions, respectively). **l**, Density scatter plots showing iN_2 E–P contact strengths. **m**, Same as **l** but for DNA methylation. **n**, Contact map (top) and aggregated accessibility of matched single-cell ATAC-seq clusters (bottom) at the Mdga1 locus. Depicted are the identified linked enhancers (arcs), colored by the Pearson correlation of the enhancer accessibility and Mdga1 expression. Dashed circles highlight dynamic E–P interactions. The shaded region highlights the *Mdga1* locus and its associated contact map (top) and the aggregated accessibility of matched single-cell ATAC-seq clusters (bottom). Statistical significance in **f**, **h**–**i** and **k** was calculated using a two-sided paired Wilcoxon rank-sum test. Obs and exp refer to observed and expected chromatin contacts, respectively. All box plots display median (line), 25th or 75th percentiles (box) and 10th or 90th percentiles (whiskers). All experiments were performed with murine cells.

**Extended Data Fig. 5:**
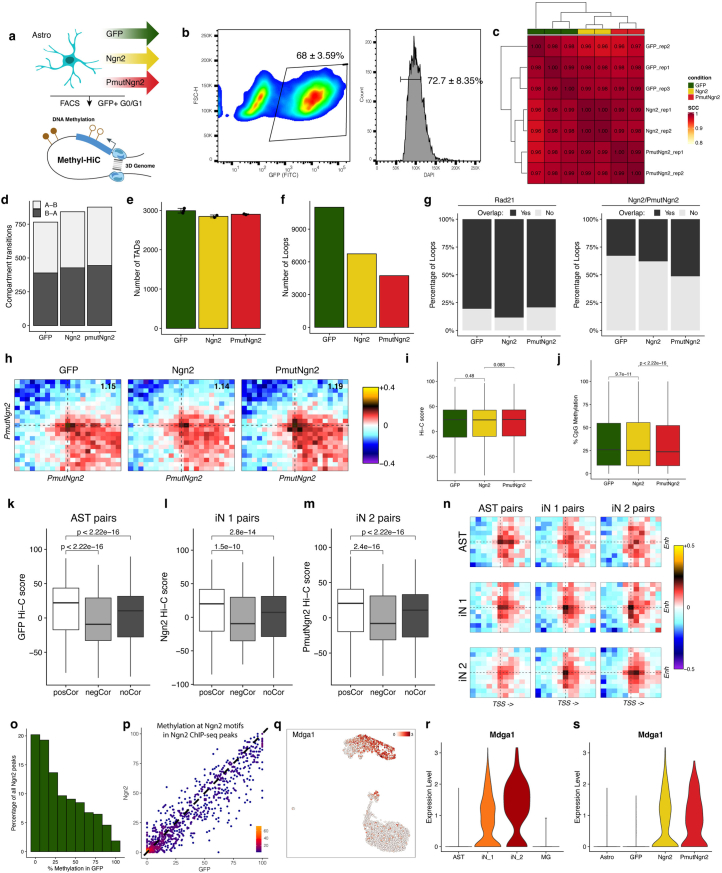
Ngn2 and PmutNgn2 rewire the 3D genome. **(a)** Schematic representation of the methyl-HiC experimental strategy **(b)** FACS gating strategy for sorting transduced (GFP + ) cells in the G_0_/G_1_ cell cycle stage. **(c)** Pairwise correlation matrixes displaying 3D genome correlation coefficient (stratum adjusted correlation coefficient, 50 kb bins, calculated by HiCRep). **(d)** Stacked bar plots depicting compartment transitions. **(e)** Barplot showing the number of TADs per condition (n = 3, 2 and 2 biological replicates for GFP, Ngn2 and PmutNgn2 respectively). Data is represented as mean ± s.d. and individual values are shown as dots. **(f)** Barplot showing the number of chromatin loops per condition (FDR < = 0.1). **(g)** Stacked barplots showing the percentage overlap between Rad21 or Ngn2/PmutNgn2 peaks and loop anchors. **(h)** Aggregated Hi-C plots between intra-TAD pairs of the top 5000 PmutNgn2 peaks. **(i)** Quantification of the interaction strength of intra-TAD contact pairs depicted in (h) (n = 7036 pairs). Statistical significance is calculated using a two-sided, paired Wilcoxon rank-sum test. **(j)** Quantification of the average DNA methylation at the distal regions within the top PmutNgn2 distal sites (n = 4141). Statistical significance is calculated using a two-sided, paired Wilcoxon rank-sum test. **(k-m)** HiC scores measured in AST, iN 1 or iN 2 between cluster-specific positive (posCor), negatively (negCor) or non-correlated (noCor) enhancer-gene pairs. Statistical significance is calculated using a two-sided, unpaired Wilcoxon rank-sum test. Number of regions per category are indicated directly at the plot. **(n)** Aggregated Hi-C maps between the linked distal peak and the transcription start site (TSS) of cluster specific enhancer-gene pairs. Genes are oriented according to transcription. **(o)** Histogram depicting the percentage of methylation in the GFP methyl-HiC condition at Ngn2 ChIP-seq binding sites. **(p)** Density scatter plot showing the level of DNA methylation at Ngn2 motifs within Ngn2 ChIP-seq peaks. **(q)** Expression levels of Mdga1 visualised on joint UMAP projection. **(r-s)** Violin plot depicting the expression levels of Mdga1 in the indicated cell-type clusters or conditions. All boxplots display median (line), 25th or 75th percentiles (box) as well as 10th or 90th percentiles (whisker).

Next, we examined DNA methylation and 3D chromatin remodeling dynamics using Methyl-HiC (Extended Data Fig. 5a–c). We observed a global re-organization of the 3D chromatin architecture, with increased short-range and decreased long-range interactions upon reprogramming (Fig. 5a,b)^22^. Remarkably, the global chromatin organization in the iNs at 2 dpi (especially in the PmutNgn2 condition; Fig. 5a, red line) resembled the profile in mouse cortical neurons in vivo^19^.

Similar to neuronal differentiation^19,22^, direct neuronal reprogramming was associated with stronger compartmentalization and increased interactions within the inactive B compartment, particularly in the PmutNgn2 condition (Fig. 5b,c and Extended Data Fig. 5d), along with increased insulation at topologically associating domain (TAD) boundaries (Fig. 5d). Although the number of TADs was unchanged (Extended Data Fig. 5e), the number of loops decreased during reprogramming, mostly in the PmutNgn2 condition (Extended Data Fig. 5f). Most of these loops were bound by cohesin, and the ratio of loops bound by Ngn2/PmutNgn2 increased with reprogramming (Extended Data Fig. 5g). The comparison of contact enrichment at pairs of Ngn2-bound sites showed that they interact more strongly during reprogramming (Fig. 5e,f). A similar trend was observed with PmutNgn2 sites (Extended Data Fig. 5h,i), with higher starting interaction strength in the GFP population, presumably due to most of the PmutNgn2 peaks corresponding to promoters.

DNA methylation levels were reduced at distal Ngn2 sites and decreased even further in the PmutNgn2 condition (Fig. 5g,h and Extended Data Fig. 5j). Interestingly, many Ngn2-bound sites were already hypomethylated in the GFP condition but became even further demethylated upon Ngn2 or PmutNgn2 binding (Extended Data Fig. 5o,p).

To address if there is global rewiring of regulatory interactions, we examined the aggregated E–P contacts for each cluster based on the previously identified EGPs (Fig. 3g). Positively correlated E–P pairs were characterized by stronger contacts (Extended Data Fig. 5k–m), with the highest contact strength observed in the cell type where the enhancer was most active and the gene was expressed (Fig. 5i and Extended Data Fig. 5n). E–P contact strength in the PmutNgn2 and the Ngn2 conditions was similar (Fig. 5i), suggesting that changes in chromatin accessibility and gene expression associated with neuronal maturation can occur independently of chromatin looping.

We observed decreased methylation levels at enhancers in iN EGPs (Fig. 5j), consistent with increased chromatin accessibility at these loci (Fig. 4a). These changes were often uncoupled—for example, astrocyte-specific enhancers also becoming further demethylated despite losing accessibility (Fig. 5j,k). In contrast to looping, enhancers became even further demethylated in the PmutNgn2 versus Ngn2 condition (Fig. 5j–m), indicating a complex relationship among chromatin accessibility, DNA methylation dynamics at enhancers and E–P loop formation upon reprogramming.

We could also identify coordinated changes at enhancer elements associated with examples such as the chromatin remodeling factor Baz1a (ref. ^49^) and the DNA-binding protein Setbp1 (ref. ^50^), implying some degree of feed-forward regulatory loops. Coordinated alterations to chromatin interactions and DNA hypomethylation were also detected for genes encoding crucial neuronal functions, such as *Kirrel3*, *Scna1*, *Plexina2* and *Auts2* as well as known neurogenic TFs—for example, *Sox11*—or TFs—for example, *Zhx3* and *Mkx* (Supplementary Table 2). Likewise, *Mdga1*, involved in cell adhesion and synapse formation in the developing brain^51,52^, was induced in direct reprogramming and upregulated during neuronal maturation (Extended Data Fig. 5q–s). *Mdga1* enhancers engaged in stronger looping with the *Mdga1* promoter, and some were also characterized by lower levels of DNA methylation in the PmutNgn2 condition (Fig. 5l–n). Notably, Ngn2-bound EGPs with a coordinated increased interaction and decreased methylation selectively in the PmutNgn2 condition comprised the neuronal maturation factor Rbfox3 (encoding for NeuN) and further epigenetic regulators, such as Kdm7a, an H3K9-demethylase and H3K27-demethylase that is involved in neural induction. These data identify a comprehensive set of genes with coordinated epigenetic remodeling in mouse astrocyte-to-neuron reprogramming.

### Ngn2 and Yy1 synergize to alter the epigenetic landscape

Given that DNA binding of PMutNgn2 could not explain its superior chromatin remodeling, we searched for potential co-factors. RNA-seq analysis identified *Yy1* (Fig. 2k), coding for a TF and epigenetic regulator that influences gene expression through the regulation of E–P loop formation^53,54^.

**Fig. 6:**
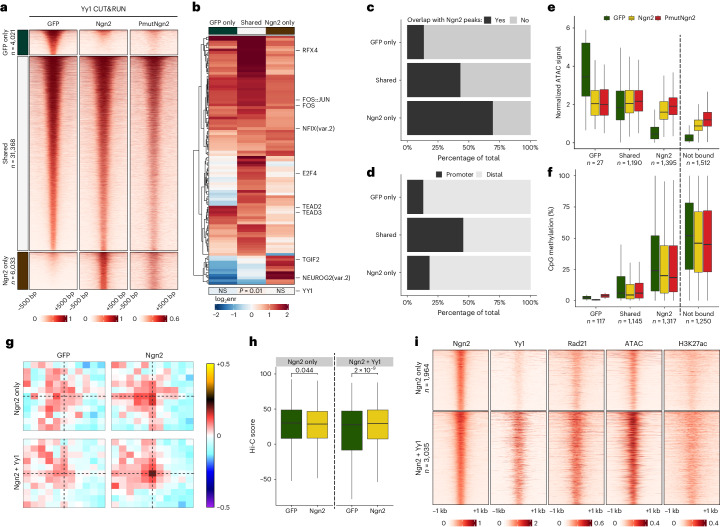
Ngn2 and Yy1 synergize to alter the epigenetic landscape. **a**, Heatmaps showing the enrichment of Yy1 CUT&RUN signal around differentially bound or shared peaks. **b**, Heatmap depicting the TF motif enrichment in the peak groups shown in **a**. **c**, Percentage overlap between Ngn2 peaks and either differentially bound or shared Yy1 peaks. **d**, Overlap of differential or shared Yy1 peaks with genomic features. **e**, Box plots depicting chromatin accessibility at the top 5,000 Ngn2 peaks overlapping differential or shared Yy1 peaks or not bound by Yy1. **f**, As in **e** but quantifying DNA methylation at the respective regions. Box plots display median (line), 25th or 75th percentiles (box) and 10th or 90th percentiles (whiskers). *n* indicates the number of regions in each category. **g**, Aggregated Hi-C plots between intra-TAD pairs of the top 5,000 Ngn2 ChIP-seq peaks, split by whether they overlap (Ngn2+Yy1) or not (Ngn2 alone) with Yy1. **h**, Quantification of the interaction strength of the pairs depicted in **g**. Statistical significance was calculated using a two-sided paired Wilcoxon rank-sum test (*n* = 1,038 and *n* = 3,035 pairs, respectively). Box plots display median (line), 25th or 75th percentiles (box) and 10th or 90th percentiles (whiskers). **i**, Heatmaps showing the enrichment of Ngn2, Yy1, Rad21, pseudobulk single-cell ATAC and H3K27ac signal around the same peaks as in **g**. Experiments were performed with murine cells. enr, enrichment; NS, not significant.

To understand if Yy1 and Ngn2 function synergistically during reprogramming, we performed Yy1 CUT&RUN in GFP and Ngn2 transduced astrocytes at 2 dpi (Fig. 6a). Although most of the sites were shared, a subset of Yy1 peaks was either lost (GFP-specific) or gained (Ngn2-specific) upon reprogramming. Because Yy1 binding was similar between the PmutNgn2 and Ngn2 conditions, we focused on comparing GFP-specific and Ngn2-specific peaks. The Yy1 motif was not enriched at the Ngn2-specific peaks, whereas the Ngn2 motif was enriched at the Yy1 peaks present only in the Ngn2 condition (Fig. 6b), suggesting that Ngn2 recruits Yy1 to these locations. Accordingly, most of the Ngn2-specific Yy1 sites were also bound by Ngn2, primarily representing distal regions (Fig. 6c,d).

Although Yy1 binding was not observed at all Ngn2 peaks with increased accessibility, its presence was associated with stronger chromatin opening (Fig. 6e). However, there was no change in DNA methylation (Fig. 6f), suggesting that Yy1 contributes primarily at the level of accessibility. Finally, co-binding of Ngn2 and Yy1 was correlated with increased chromatin looping (Fig. 6g,h) as well as increased binding of Rad21 and the accumulation of H3K27ac (Fig. 6i).

Overall, our results identify Yy1 as a putative Ngn2 co-factor that facilitates chromatin opening, recruitment of cohesin and looping at co-bound regions.

### Yy1 is required for successful direct neuronal reprogramming

**Fig. 7:**
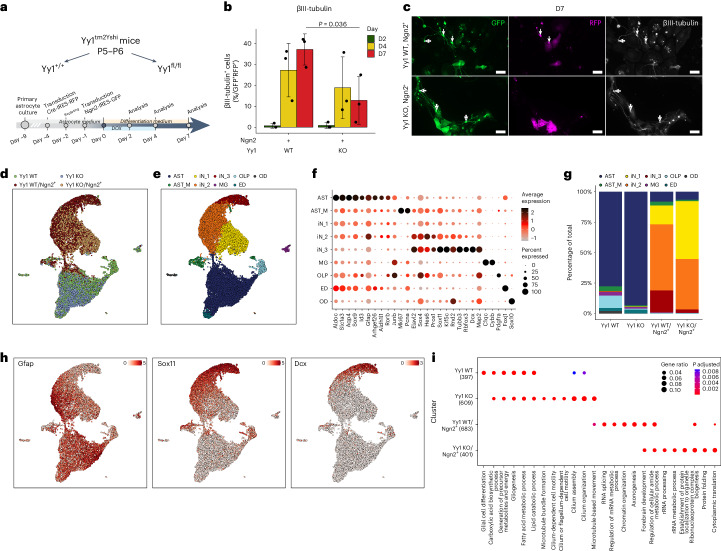
Successful murine glia-to-neuron reprogramming requires Yy1. **a**, Scheme of the experimental design. **b**, Quantification of the proportion of GFP^+^RFP^+^ immunopositive for βIII-tubulin. Data are plotted as mean ± s.d.; each dot represents an individual biological replicate (*n* = 3). Statistical significance was calculated using a linear regression model. **c**, Representative micrographs of immunocytochemistry as indicated at 7 dpi of Ngn2 (GFP^+^) in the indicated conditions. The filled arrow indicates βIII-tubulin^+^ iNs in the double-transduced (GFP^+^RFP(Cre)^+^) cells; the empty arrow indicates βIII-tubulin^+^ iNs transduced only with Ngn2 (GFP^+^). Scale bar, 50 µm. Immunostainings were independently replicated (*n* = 3). **d**, Single-cell RNA-seq UMAP projection of the indicated experimental conditions. **e**, Same as **d** but for the identified cell type clusters. **f**, Dot plot showing the proportion of cells (as a percentage, size of dots) and the expression levels of selected marker genes in respective cell type clusters. **g**, Stacked bar plot depicting the proportion of the cell types in the respective experimental conditions. **h**, UMAP visualization of the expression of the indicated marker genes. **i**, Bubble plot depicting the GO term enrichment in the indicated experimental conditions. D, day; KO, knockout; WT, wild-type. Source data

To functionally test the role of Yy1 in neuronal reprogramming, we deleted it in primary cortical astrocytes derived from Yy1^*tm2Yshi*^ homozygous mice by using a Cre-expressing lentivirus followed by Ngn2 expression (Fig. 7a). At 7 dpi, approximately 13% of iNs (βIII-tubulin^+^/GFP^+^RFP^+^) were observed in the Yy1 KO/Ngn2^+^ condition, thus significantly lower than 37.1% iNs in the Yy1 WT/Ngn2^+^ condition (Fig. 7b,c).

**Extended Data Fig. 6:**
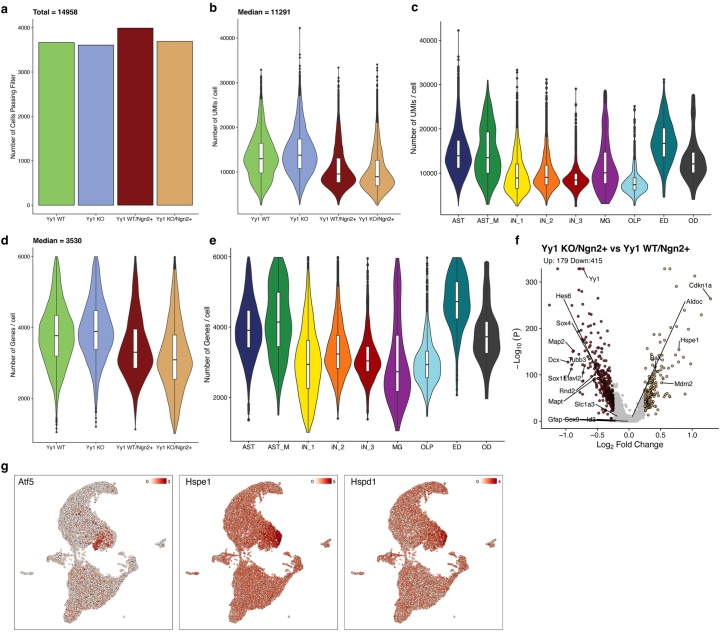
Yy1 is required for successful mouse astrocyte-to-neuron conversion. **(a)** Bar plot showing the number of cells per experimental condition that passed the quality control filters and were used for downstream analysis. **(b)** Violin and box-whisker plot depicting the number of UMIs per cell for each experimental condition (n = 3666, 3608, 3991 and 3693 cells per condition respectively). **(c)** Same as d, but for each annotated cell-type cluster (n = 6790, 338, 2378, 3691, 835, 175, 444, 208 and 99 cells per cluster respectively). **(d)** Violin and box-whisker plot depicting the number of genes per cell for each experimental condition (n = 3666, 3608, 3991 and 3693 cells respectively). **(e)** Same as f, but for each annotated cell-type cluster (n = 6790, 338, 2378, 3691, 835, 175, 444, 208 and 99 cells per cluster respectively). **(f)** Volcano plot showing the differentially expressed genes for the following pairwise comparison, Yy1_KO/Ngn2+ vs Yy1_WT/Ngn2. Yellow, red, and grey dots represent the up-, down- and non-regulated genes. **(g)** UMAP visualization of the expression levels of the indicated marker genes. All boxplots display median (line), 25th or 75th percentiles (box),10th or 90th percentiles (whisker) and outliers (dots).

To elucidate the molecular mechanisms underlying the impaired reprogramming in Yy1_KO astrocytes, we performed single-cell RNA-seq (Extended Data Fig. 6a–e). Distinct iN clusters were associated with increased neuronal and decreased astrocyte gene expression (Fig. 7d–g). Cluster iN_1 had partially downregulated genes associated with the original astrocyte identity and weakly upregulated genes associated with an early pan-neuronal program (for example, *Sox4*, *Hes6* and *Sox11*)^4^, whereas, in cluster iN_2, the latter was clearly upregulated (Fig. 7d–h). Cluster iN_3 represented the most mature iN cluster, with strongest downregulation of astrocyte genes and upregulation of neuronal genes, such as *Dcx* and *Rbfox3* (Fig. 7f–h). Remarkably, most Yy1 KO cells were located in the unstable iN_1 and less mature iN_2 state (Fig. 7g). This correlated with the decreased expression of neuronal markers (for example, *Dcx, Sox11, Rnd2* and *Map2*; Extended Data Fig. 6f). Thus, *Yy1* deletion leads to early deficits in neuronal reprogramming.

GO analysis on the top 200 differentially expressed genes for each experimental condition revealed ‘forebrain development’ and ‘axonogenesis’ as terms enriched in the Yy1 WT/Ngn2^+^ iNs, indicative of an ongoing conversion process (Fig. 7i and Supplementary Table 3). Conversely, GO terms such as ‘rRNA processing’ and ‘protein folding’ suggested that the lack of Yy1 is associated with hallmarks of an integrated stress response (including *Atf5*, a stress-responsive TF, and mitochondrial chaperones, such as *Hspe1* and *Hspd1*; Extended Data Fig. 6f,g), which could present a hurdle in direct reprogramming (Fig. 7i)^29,55,56^. These GO terms were not enriched in the Yy1 KO astrocytes or the control clusters (Fig. 7i), indicating the specificity for the reprogramming condition without Yy1.

Thus, the failure of direct mouse neuronal reprogramming upon *Yy1* deletion is characterized by the incomplete induction of the neuronal program and activation of a protein stress response.

### Yy1 strengthens Ngn2-mediated epigenetic remodeling

**Fig. 8:**
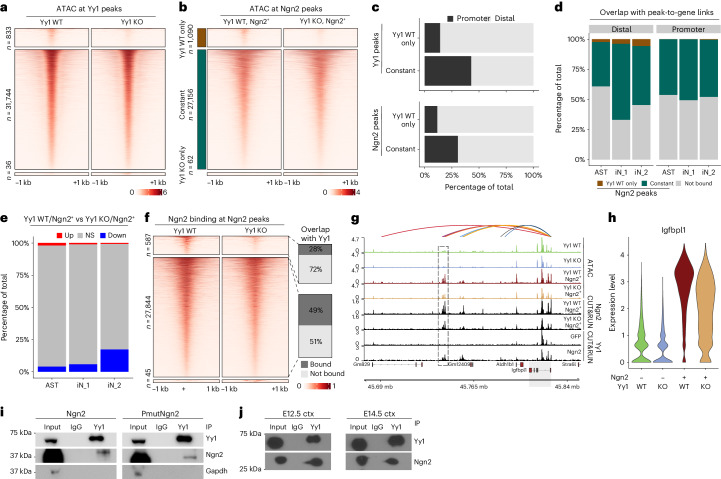
Yy1 facilitates Ngn2-mediated epigenetic remodeling. **a**, Heatmaps showing the enrichment of chromatin accessibility signal around differentially bound or shared Yy1 peaks in astrocytes from Yy1 WT or Yy1 KO mice, respectively. **b**, Heatmaps showing the enrichment of chromatin accessibility signal around differentially accessible or shared Ngn2 peaks in either Yy1 WT+Ngn2 or Yy1 KO+Ngn2 condition. **c**, Overlap of Yy1 and Ngn2 peaks that lose accessibility (Yy1 WT) or remain unchanged (shared) with genomic features. **d**, Overlap of Yy1 and Ngn2 peaks that lose accessibility or remain unchanged with EGP links as identified in Fig. 3g. **e**, Percentage of differentially regulated genes (based on single-cell RNA-seq) overlapping with either distal or promoter EGP anchor. **f**, Heatmaps showing Ngn2 (Flag) CUT&RUN enrichment at either differentially bound or shared peaks. Shown is also the ratio between peaks overlapping with Yy1 in each category. **g**, Genome track showing the linked enhancers (arcs), chromatin accessibility, Ngn2 and Yy1 CUT&RUN signal in the indicated conditions at the *Igfbpl1* locus. A cluster of enhancers with reduced accessibility upon Yy1 KO is indicated with a dashed rectangle. The shaded region highlights the *Igfbpl1* locus. **h**, Violin plot depicting the expression levels of *Igfbpl1* in the indicated conditions. **i**, Co-IP experiments in P19 cells showing pulldown using IgG or Yy1 antibody and staining for Yy1, Ngn2 and Gapdh. **j**, Similar to **i** but using either E12.5 or E14.5 cortex with endogenous Yy1 and Ngn2. **i**,**j**, Co-IP experiments were independently replicated (*n* = 2). ctx, cortex; KO, knockout; NS, not significant; WT, wild-type. Source data

**Extended Data Fig. 7:**
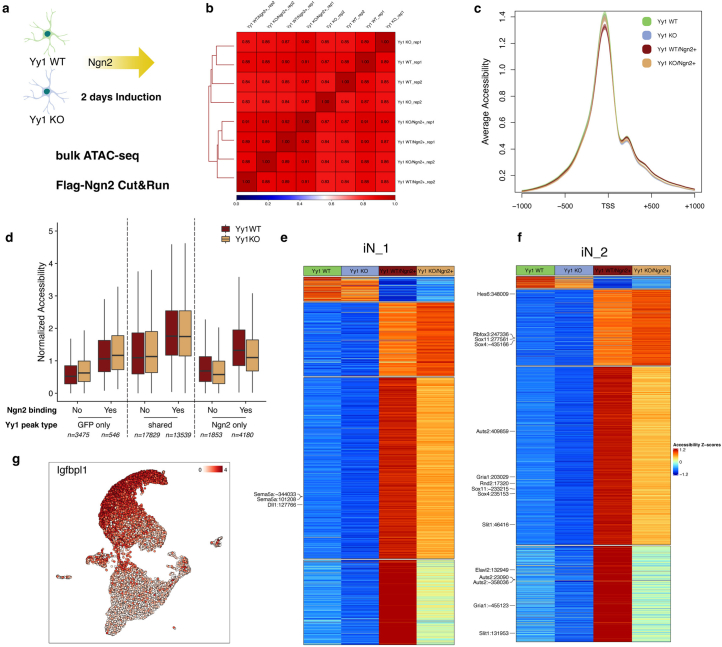
Yy1 deletion impairs Ngn2-mediated chromatin remodelling. **(a)** Schematic representation of the experimental setup **(b)** Pearson’s correlation for ATAC in the indicated experimental conditions (N = 2 biological replicates). **(c)** Average accessibility (+/- 1 kb) at all TSSs in the indicated experimental conditions. **(d)** Boxplots depicting the normalized accessibility levels in Yy1WT/KO + Ngn2 conditions at different categories of Yy1 peaks (based on Fig. 7a) grouped by the overlap with Ngn2 binding sites. Boxplots display median (line), 25th or 75th percentiles (box),10th or 90th percentiles (whisker) and outliers (dots) **(e-f)** Heatmaps depicting the accessibility levels at iN_1 or iN_2 distal enhancers in the indicated experimental conditions. **(g)** UMAP visualization of the expression levels of Igfbpl1.

To elucidate the role of Yy1 in consolidating Ngn2-mediated chromatin remodeling at iN enhancers, we performed bulk ATAC-seq in Yy1 WT and Yy1 KO conditions at 2 dpi (Extended Data Fig. 7a–c). Less than 2.6% of the Yy1 peaks were associated with reduced accessibility, suggesting that Yy1 is not necessary to maintain chromatin accessibility (Fig. 8a). Likewise, most Ngn2 peaks remained accessible despite lack of Yy1 (Fig. 8b). As expected, we observed that, for both Yy1 and Ngn2 peaks, the sites sensitive to Yy1 loss represent primarily distal regions (Fig. 8c), in particular the Ngn2-specific Yy1 peaks previously identified (Fig. 6a,b and Extended Data Fig. 7d). Furthermore, they were more enriched in iN than astrocyte enhancers (Fig. 8d). The accessibility at most iN distal regions was reduced upon Yy1 KO (Extended Data Fig. 7e,f), and iN_2 genes were more downregulated than genes belonging to other clusters (Fig. 8e).

To address if Yy1 is required for Ngn2 binding, we performed CUT&RUN. We found that almost all of the sites remained bound by Ngn2, even in the absence of Yy1 (Fig. 8f), suggesting that Yy1 acts downstream of Ngn2 and is required for the activation of a small, but important, subset of neuronal enhancers. For example, Yy1 deletion led to a decrease in chromatin accessibility at some enhancers predicted to regulate *Igfbpl1* (insulin-like growth factor binding protein-like 1), a gene implicated in regulation of axon elongation^57^ (Fig. 8g and Extended Data Fig. 7g). This was accompanied by a downregulation of *Igfbpl1* expression, despite no obvious change in Ngn2 binding (Fig. 8g,h).

To query possible direct Yy1–Ngn2 interaction, we performed co-immunoprecipitation (IP) in P19 cells. Indeed, Yy1 was able to pull down both Ngn2 and PmutNgn2 (Fig. 8i). To exclude possible overexpression artifacts, we also performed co-IP experiments using endogenous proteins in the E12.5 or E14.5 mouse developing cortex and observed similar results (Fig. 8j).

Thus, we identified Yy1 as a direct interactor of Ngn2 that aids chromatin remodeling at neuronal enhancers and boosts the associated transcriptional output. Yy1 is not necessary for Ngn2 binding but is recruited by Ngn2, and its absence impairs the activation of some Ngn2 targets and, thereby, neuronal reprogramming.

## Discussion

In this study, we gained several mechanistic insights into how to improve murine glia-to-neuron reprogramming—by exploring the phosphomutant form of Ngn2 and by identifying a direct interactor of Ngn2, namely Yy1.

Our phenotypic and molecular data demonstrate that PmutNgn2 is a superior reprogramming factor based on a faster neuronal conversion rate and improved iN maturation, as demonstrated by several methods. The PmutNgn2-associated transcriptome is characterized not only by the upregulation of more neuronal maturation genes but also by a stronger downregulation of astrocyte identity genes. These data highlight the importance of erasing the initial cell identity to further progress along a new fate trajectory^6^. The silencing of astrocyte genes is partially a direct effect, as we found evidence for Ngn2 binding at these enhancers and regulating gene sets associated with gliogenic function, possibly by sequestering transcriptional co-factors, such as CBP and Smad1, away from gliogenic promoters^58,59^. Dephosphorylation of Ngn2 improves its interaction with other E-box binding proteins^25,47,60^: indeed, Tcf3 motifs are more accessible in the PmutNgn2 condition, thus enhancing the expression of pro-neurogenic determinants.

Based on epigenetic profiling, PmutNgn2 activated more neuronal genes and had a higher efficiency in inducing DNA demethylation than Ngn2. This is intriguing as PmutNgn2 promotes predominantly the maturation of the iNs, supporting the concept that DNA methylation is more relevant for neuronal maturation than fate restriction^16^. PmutNgn2 also augmented chromatin looping on regulated EGPs and increased active enhancer marks. However, these differences could not be explained by DNA binding, as the sites bound specifically by PmutNgn2 were predominantly open promoter sites and did not contain any notable specific motifs, and bound genes only mildly increased in expression. Apparently, the pioneering activity of PmutNgn2 is not improved compared to Ngn2 (ref. ^47^). This is of interest, as protein levels may differ between Ngn2 and its dephosphorylated form^25^. However, PmutNgn2-specific peaks would be difficult to explain by higher protein levels alone.

We further identified a co-factor of Ngn2, Yy1 (ref. ^54^), whose interaction with Ngn2 correlates with increased chromatin looping. CUT&RUN experiments demonstrated Yy1 recruitment by Ngn2, as Ngn2-specific Yy1 binding sites contained Ngn2 motifs. Conversely, Ngn2 binding was not altered when Yy1 was deleted, suggesting that Yy1 is not required to recruit or maintain Ngn2 at its target sites. Distal regions of neurogenic genes bound by Ngn2 and expressed in iNs were most sensitive to Yy1 loss (Fig. 8c), which could explain why the initial stages of neuronal conversion could occur in the absence of Yy1, whereas the progression to the stages associated with higher neurogenic gene expression and the shutdown of astrocyte gene expression were impaired. Intriguingly, Yy1 KO led to the induction of a stress response and ultimate failure of direct neuronal reprogramming compared to controls. Notably, in Yy1 KO, several chromatin remodelers, such as *Baz1b*, *Brpf1* and *Sox4*, became neither accessible nor upregulated, which may contribute to the failure to further increase iN gene expression. Importantly, the gene network regulated by Yy1 in reprogramming is very different from its developmental role, where it exerts a key role as metabolic regulator^41^. However, effects on metabolism or proteostasis may well contribute to indirect effects that Yy1 may have in reprogramming. Thus, Yy1 directly interacts with Ngn2, acting as an adaptor protein to promote the activation of neurogenic genes and downregulation of the astrocyte fate. These data call for searching additional co-factors of proneural TFs, involved in astrocyte-to-neuron reprogramming.

In conclusion, we not only identified how direct mouse neuronal reprogramming is associated with coordinated multi-scale epigenetic remodeling, but we also revealed that co-factors, such as Yy1, are key contributors to fate conversion by these proneural factors. These findings not only further our understanding of how a single TF can rewire several epigenetic layers but also pave the way toward removing reprogramming roadblocks and improving the generation of more mature neurons.

## Methods

### Transgenic mice

All experimental procedures for this study were performed at the Biomedical Center, LMU Munich, in accordance with German and European Union guidelines and were approved by the government of Upper Bavaria. Primary cultures of mouse astrocyte were obtained from the cortex of R26-M2rtTA and *Yy1*^*tm2Yshi*^ (ref. ^61^) mice of P5–6 days of age. R26-M2rtTA (no. 006965) and *Yy1*^*tm2Yshi*^ (no. 014649) mice were obtained from The Jackson Laboratory. The mice were not selected based on their gender. The mice were fed ab libitum; housed in individually ventilated cage systems in a room with a temperature of 22 °C ± 2 °C, 55% ± 10% humidity and a 12-h/12-h light/dark cycle; and maintained under specific pathogen-free conditions.

### Primary mouse astrocyte cultures

Astrocytes were isolated^4,32^ by dissecting three postnatal mice (P5–6), and both the gray and white matter of the cerebral cortex were isolated, after removing the subventricular zone, striatum and hippocampus. The cortical meninges were also removed. The cortical tissue was mechanically dissociated, and the cell suspension was centrifuged at 300*g*, 4 °C, for 5 min. The cell pellet was resuspended in astrocyte medium consisting of DMEM/F12 (1:1) with GlutaMAX (Thermo Fisher Scientific), 10% FBS, penicillin–streptomycin (Gibco), glucose (Gibco), 1× B27 serum-free supplement (Gibco), 10 ng ml^−1^ epidermal growth factor (EGF, Gibco) and 10 ng ml^−1^ basic fibroblast growth factor (bFGF, Gibco). The resulting cell suspension was plated onto a T-25 flask. The primary astrocyte culture was maintained in an incubator for 7 d at 37 °C and 5% CO_2_. Thereafter, the cells were passaged using 0.05% trypsin/EDTA (Thermo Fisher Scientific) and plated onto the following poly-d-lysine (PDL) (Sigma-Aldrich) coated surfaces for the following experiments: 50,000 cells per well in a 24-well plate in 500 µl of media for immunocytochemistry; 200,000 cells per six-well plate for bulk-RNA-seq, bulk-ATAC-seq, 10x multiome and 10x single-cell RNA-seq experiments; and 1,000,000 cells per T-25 flask for ChIP-seq.

### Plasmids

The plasmid FUW-TetON was modified to insert Gateway cloning sites. Mouse Ngn2, eGFP and 9S-A Ngn2 (referred to as PmutNgn2, which was a gift from A. Philpott)^25^ were cloned into the Gateway entry vectors (Thermo Fisher Scientific) and subsequently shuttled into the dox-inducible lentiviral expression vector FUW-TetON by employing Gateway recombination cloning technology (Thermo Fisher Scientific). The lentiviral expression vector was characterized by the presence of a tetracycline response element followed by the mammalian CMV2 promoter, which regulated the expression of the TFs and the eGFP (fluorescent reporter employed to identify transduced cells). The TF sequence was separated from the eGFP sequence by an internal ribosome entry site (IRES).

### Lentiviral production and viral titer determination

Vesicular stomatitis virus-glycoprotein (VSV-G)-pseudotyped lentiviral particles were produced by transfecting 293T cell line with the following plasmids: pCMVdR8.91 (expressing gag, pol and rev genes), pVSVG and lentiviral expression plasmid. The lentiviral particles were harvested and concentrated by ultracentrifugation at 125,000*g* for 2 h, and the pellet containing the lentiviral particles was resuspended in 1× PBS (supplemented with 5 mM MgCl_2_). The lentivirus was aliquoted and stored at −80 °C until use. The lentiviral titer was determined by a functional assay, where primary mouse astrocytes were infected with the lentivirus preparation at various dilutions, and the number of successfully infected cells was determined by immunostaining the transduced cells with an anti-GFP antibody (for TF-encoding lentiviruses) or an anti-RFP antibody (for Cre-expressing lentivirus). The viral titers used in all the experiments were in the range of 10^10^ to 10^12^ transducing units per milliliter.

### Viral transduction of primary mouse astrocytes

After seeding the desired number of cells in PDL-coated plates, 24 h later the cells were transduced with 10^7^ to 10^9^ transducing units per microliter of lentiviral particles. Approximately 20 h after transduction, the astrocyte medium was replaced with fresh medium containing DMEM/F12 (1:1), supplemented with penicillin–streptomycin, glucose, 1× B27 and GlutaMAX (differentiation medium), and the cells were maintained in culture in a 9% CO_2_ incubator for a period, depending upon the experimental design. To induce the expression of the TF and fluorescent protein, dox (2 μg ml^−1^) was added to the differentiation medium, and the dox-containing medium was added freshly for four consecutive days.

### Fluorescence-activated cell sorting

Cells were prepared for fluorescence-activated cell sorting (FACS) by washing them once with 1× PBS followed by trypsinization (0.05% trypsin in EDTA) for 5 min. The trypsinization reaction was stopped by adding astrocyte medium. The harvested cells were then washed twice with ice-cold PBS and centrifuged at 300*g* for 3 min at 4 °C. The cells were resuspended in DMEM/F-12 (1:1), and a single-cell suspension was generated using a 40-μm cell strainer. FACS was performed by employing a FACSAria Fusion (BD Biosciences) using a 100-μm nozzle. The gating strategy was set by using forward, side scatter and untransduced astrocytes as a negative control and eGFP-expressing astrocytes as a positive control. Additionally, for Methly-HiC, astrocytes were stained for DAPI, and only cells in G_0_ and G_1_ (single DNA content) were sorted. The cells were sorted into DMEM/F-12 (1:1).

### Immunocytochemistry

Coverslips containing astrocytes were fixed using 4% paraformaldehyde in 1× PBS for 10 min at room temperature. The cells were washed twice with 1× PBS and stored for up to 3 weeks at 4 °C before staining. The coverslips were incubated with blocking solution (3% BSA, 0.5% Triton X-100 in 1× PBS) for 30 min. Thereafter, the coverslips were incubated with the primary antibody diluted (for detailed information about antibodies used, see Supplementary Table 4) using blocking solution overnight at 4 °C. After washing the coverslips three times with 1× PBS, they were incubated with the appropriate secondary antibody (diluted 1:500) for 1 h at room temperature. The coverslips were stained with DAPI (diluted 1:1,000 in blocking solution) for 10 min at room temperature. Finally, the coverslips were mounted using Aqua-Poly/Mount (Polysciences).

### Live-imaging microscopy

A Zeiss Cell Observer was employed to perform continuous live imaging of astrocyte-to-neuron conversion. The acquisition of images was performed as follows. Phase contrast images and fluorescent images (GFP) were captured every 20 min and 4 h, respectively, with a ×10 phase contrast objective (Zeiss) and an AxioCam HRm camera. Zeiss AxioVision 4.7 software was controlled by a custom-made VBA module (TAT, Timm Schroeder, ETH Zürich)^62^. The movie processing and analysis was performed in ImageJ (1.53q) (National Institutes of Health).

### Quantification and statistical analysis of immunocytochemistry and live imaging

The acquisition of microscopy images was performed using an AxioM2 epifluorescence microscope (Zeiss) or an LSM 710 laser scanning confocal microscope (Zeiss) and ZEN2 software (version 2.0.0.0, Zeiss). The quantification of iNs was performed by applying the following stringent criteria, which were previously described in Gascon et al.^5^. iNs had to possess a unipolar or bipolar morphology, with a process being at least three times the length of its soma. Additionally, the iNs had to be βIII-tubulin positive and GFAP negative. In case of the live-imaging microscopy, the time of conversion was defined as a timepoint (in hours) when a GFP^+^ cell acquired neuronal morphology—that is, exhibited a unipolar or bipolar morphology where the process was at least three times the length of its soma. Statistical analysis was performed in R (version 4.2.1). In Figs. 1e–h,j and 7c and Extended Data Fig. 1e, statistical significance was calculated with linear regression by implementing the function ‘lm’ in RStudio on log_2_-transformed reprogramming rate^14^.

### Statistics and reproducibility

The primary astrocytes, transduced with the GFP, Ngn2 or PmutNgn2 lentivirus, were obtained from the same litter of mice. In case of the primary astrocytes obtained from the Yy1^*tm2Yshi*^ line for the functional studies (conditional knockouts of the candidate gene, Yy1), the wild-type, heterozygote and homozygote genotypes were obtained from same litter of mice by crossing two heterozygote mice.

No statistical methods were used to pre-determine samples sizes, but our sample sizes relied on previous experience, showing that this sample size gives sufficient statistical power^5,6,17–19,45,63^. No data were excluded from the analyses. For data in Figs. 1e–h,j and 7c, the values were log transformed and, hence, assumed to be normally distributed.

All the data analysis for immunocytochemistry (Figs. 1e–h and 7c) and live imaging (Fig. 1j) was blinded. The genomic experiments and associated data analysis were not blinded because they did not involve subjective measurements.

### Intracellular DAPI staining

For the Methyl-HiC experiment, the cells were stained with DAPI following the intracellular staining protocol with the following modifications^19^. Upon fixing with 1% formaldehyde and permeabilizing the cells, they were stained with DAPI (1:1,000 dilution in wash buffer containing 1% BSA, 0.1% RNasin plus RNase inhibitor (Promega) in PBS). The cells were washed once with the wash buffer and subsequently resuspended in PBS with 1% BSA and 1% RNasin plus RNase inhibitor, filtered through a 40-μm cell strainer and FACS sorted.

### RNA extraction and bulk RNA-seq library preparation

Approximately 30,000 events per condition were FACS sorted into DMEM/F-12 (1:1) and centrifuged at 300*g* for 5 min at 4 °C. Then, the cell pellet was resuspended in TRIzol (Thermo Fisher Scientific) and further processed with an RNA Clean & Concentrator Kit (Zymo Research) to extract the RNA. The quality of the extracted RNA was determined using an Agilent RNA 6000 Pico Kit and an Agilent 2100 Bioanalyzer system. All the samples used for library preparation had an RNA integrity number (RIN) value > 8.

Next, 50 ng of RNA was used as the input material for library generation, and the protocol was a bulk adapted version of mcSCRB-seq^64,65^. cDNA was generated from the poly(A)-enriched RNA fraction using oligo-dT primers and a Maxima First Strand cDNA Synthesis Kit (Thermo Fisher Scientific). The unincorporated primers were digested using Exonuclease I (Thermo Fisher Scientific). The resulting cDNA was pre-amplified using Terra polymerase (Takara Bio). The quality of the cDNA was determined using the Agilent 2100 Bioanalyzer system. The RNA-seq library was prepared using a NEBNext Ultra II FS DNA Library Kit for Illumina (New England Biolabs) according to the manufacturer’s instructions. The quality of the RNA-seq libraries was assessed using the Agilent 2100 Bioanalyzer system.

### Bulk ATAC-seq

Bulk ATAC-seq libraries were generated by following the OMNI-ATAC-seq protocol^66^. Approximately 70,000 events were FACS sorted into tubes containing DMEM/F-12 (1:1) and centrifuged at 300*g* for 5 min at 4 °C, and the cell pellet was resuspended in ATAC resuspension buffer. The cell viability and cell number were determined using a Countess automated cell counter (Thermo Fisher Scientific). Fifty thousand viable cells were used for the Tn5 transposition reaction. The transposition reaction was performed at 37 °C for 30 min in an Eppendorf thermomixer. The transposed fragments were purified using a DNA Clean & Concentrator-5 Kit (Zymo Research). The purified transposed DNA fragments were amplified using NEBNext Ultra II Q5 Master Mix (New England Biolabs) and cleaned up using the DNA Clean & Concentrator-5 Kit. The quality of the ATAC-seq libraries was assessed using the Agilent 2100 Bioanalyzer system.

### ChIP-seq

The ChIP-seq protocol was adapted from a previously described protocol^67^. In brief, 4 million astrocytes were fixed using 1% methanol-free formaldehyde (Thermo Fisher Scientific) at room temperature for 10 min. The cross-linking reaction was terminated by the addition of 125 mM glycine followed by an incubation step at room temperature for 5 min. The cells were lysed by suspension in a hypotonic buffer (20 mM Tris, pH 7.4; 2 mM MgCl_2_; 5% glycerol; 0.6% NP-40) and incubation on ice for 5 min with mild vortexing every 30 s, which resulted in the release of the nuclei. The nuclei were resuspended in ChIP lysis buffer (20 mM Tris, pH 7.4; 150 mM NaCl; 1% sodium deoxycholate; 0.1% SDS; 1 mM EDTA, pH 8.0) and sonicated using a Bioruptor Pico sonicator (Diagenode) with the following settings: 30 s ON/OFF, 20 cycles. The sonicated chromatin was quality controlled using the Agilent 2100 Bioanalyzer system. The sonicated chromatin used for ChIP-seq ranged from 150 bp to 300 bp.

The chromatin was pre-cleared using Dynabeads Protein G (Thermo Fisher Scientific). After pre-clearing, 10% of the pre-cleared chromatin was set aside as the input fraction. The chromatin was incubated with 4 μg of mouse monoclonal anti-FLAG M2 antibody (Sigma-Aldrich) overnight at 4 °C on a rotating wheel (10 r.p.m.). After the ChIP, Dynabeads Protein G (Thermo Fisher Scientific) was added to the ChIP sample and incubated at 4 °C for 3 h on a rotating wheel (10 r.p.m.). The ChIP sample was washed five times with LiCl was buffer (50 mM Tris, pH 7.4; 1 mM EDTA, pH 8; 1% NP-40; 1% sodium deoxycholate; 0.5 M LiCl) followed by a single wash with TE buffer (10 mM Tris, pH 8; 1 mM EDTA, pH 8). All the wash steps were performed for 5 min at 4 °C on a rotating wheel (10 r.p.m.). The elution of the protein–DNA complex was performed using the elution buffer (50 mM NaHCO_3_, 1% SDS) under the following condition: constant agitation on a thermomixer (Eppendorf) at 60*g* for 15 min at 65 °C. The eluted DNA was de-crosslinked by the addition of 5 M NaCl (final concentration: 210 mM) and incubated overnight (not more than 15 h) at 65 °C.

The de-crosslinked DNA was treated with RNase A (Thermo Fisher Scientific) and incubated in a thermomixer (Eppendorf) at 60*g* for 90 min at 37 °C, followed by treatment with Proteinase K (Ambion) and incubated in a thermomixer (Eppendorf) at 800 r.p.m. for 120 min at 55 °C. The DNA was extracted using UltraPure Phenol:Choloroform:Isoamylalcohol (25:24:1, v/v, Thermo Fisher Scientific) following the manufacturer’s instructions and precipitated by ethanol precipitation (glycogen, 3 M sodium acetate, pH 5.2, 100% ethanol) overnight at −20 °C. The DNA was resuspended in low TE buffer and quantified Qubit dsDNA HS (Thermo Fisher Scientific). One nanogram of ChIP DNA was used as starting material for library preparation with the MicroPlex Library Preparation Kit v2 (Diagenode). The quality of the ATAC-seq libraries was assessed using the Agilent 2100 Bioanalyzer system.

### CUT&RUN

The Yy1, FLAG, Rad21 and H3K27Ac CUT&RUN assays were performed as previously described with specific modifications^63^.

In brief, 2–3.6 × 10^5^ iNs were harvested, washed twice and resuspended in wash buffer (20 mM HEPES, pH 7.5; 150 mM NaCl; 0.5 mM spermidine; 1× Roche cOmplete). Concavalin A beads (BioMag Plus, Polysciences) were activated with bead activation buffer (20 mM HEPES, pH 7.9; 10 mM KCl; 1 mM CaCl_2_; 1 mM MnCl_2_). Cells were incubated with 10 μl of activated beads for 10 min at room temperature. After incubation, the beads were resuspended in a cold antibody buffer (2 mM EDTA in digitonin buffer) containing antibody (5 μg of Yy1 (D5D9Z) rabbit monoclonal antibody 46395, 2 μg of FLAG antibody (Sigma-Aldrich, F3165-.2MG), 5 μg of Rad21 (BIOZOL, GTX106012) and 1 μg of H3k27Ac (Abcam, 39133)), and the mixture was incubated on a nutator overnight at 4 °C.

On the next day, the beads were washed twice and resuspended in 0.75 μl of pAG-Mnase in digitonin buffer (0.1% digitonin, Thermo Fisher Scientific, in wash buffer) and incubated for 10 min at room temperature on a rotator. Later, beads were washed twice with cold digitonin buffer and then resuspended in 50 μl of digitonin buffer containing 1 μl of 100 mM CaCl_2_. The suspension was incubated for 2 h at 4 °C on a nutator. After the incubation, 33 μl of STOP buffer (340 mM NaCl, 20 mM EDTA, 4 mM EGTA, 50 μg ml^−1^ RNase A (Thermo Fisher Scientific), 50 μg ml^−1^ glycogen) was added to each reaction, and the mixture was incubated for 30 min at 37 °C.

DNA extraction was performed using UltraPure Phenol:Chloroform:Isoamyl Alcohol (25:24:1, v/v, Thermo Fisher Scientific) and precipitated with 100% ethanol, 1 μl of glycogen and 1/10th volume of 3 M sodium acetate for 4–16 h at −20 °C. DNA was then dissolved in 10 μl of 1 mM Tris-HCl, pH 8, and 0.1 mM EDTA.

CUT&RUN libraries were prepared with an NEBNext Ultra II DNA Library Prep Kit for Illumina using 6–30 ng of fragmented DNA. The quality of the CUT&RUN libraries was evaluated using the Agilent 2100 Bioanalyzer system.

### Single-cell multiome ATAC + gene expression

Single-cell multiome (version 1, 10x Genomics) libraries were generated according to the manufacturer’s instruction manual. In case of the multiome libraries, we targeted for the recovery of 500 nuclei for the GFP, Ngn2 and PmutNgn2 conditions and 5,000 nuclei for the Astro condition.

### Methyl-HiC

A modified Methyl-HiC was performed^19^ based on previously described protocols^17,18^. Full details of the experimental steps can be found at https://www.protocols.io/view/methylhic-bif2kbqe/.

Pellets from frozen, fixed and FACS-sorted G_0_/G_1_ cells were thawed and then lysed on ice with 0.2% Igepal-CA630 (Sigma-Aldrich). Nuclei were subsequently permeabilized with 0.5% SDS and chromatin digested with DpnII (New England Biolabs) at 37 °C overnight. DpnII was heat inactivated at 62 °C, and then sticky ends were filled in with biotin-14-dATP (Life Technologies) before proximity ligation with T4 Ligase (New England Biolabs). Proteinase K (New England Biolabs) and NaCl was used for reverse crosslinking nuclei overnight at 68 °C, and DNA was afterward purified using ethanol precipitation. A Covaris S220 sonicator was next used to shear the DNA to approximately 550-bp fragments.

End repair was performed on the sonicated DNA with T4 DNA Polymerase (New England Biolabs). Approximately 0.01% of methylation controls were spiked into sample, and the reaction was bisulphite converted using an EZ DNA Methylation-Gold Kit (Zymo Research). Libraries were prepared using an Accel-NGS Methyl-Seq DNA Library kit (Swift Biosciences) according to the manufacturer’s instructions until the adaptor ligation step. At this point, streptavidin T1 beads (Thermo Fisher Scientific) were used for biotin pulldown of DNA, followed by stringent washes. Final libraries were amplified from the streptavidin beads using EpiMark Hot Start Taq (New England Biolabs) with Methyl-Seq indexing primers (Swift Biosciences), followed by size selection with 0.6× AMPure XP beads (Agencourt).

### P19 cell transfection

P19 cells were plated in 10-cm dishes. Cells were transfected using Lipofectamine 3000 with 5 µg of Control (Pcig2), Neurogenin2 and Neurogenin2 mutated (S-A9 TA1) DNAs and were harvested after 24 h by cell scraping using cold PBS followed by centrifugation at 300*g* for 5 min to collect the cell pellets.

### Protein extraction

The P19 cell pellets were thawed on ice and resuspended in 1× pelleted cell volume of the lysis buffer A (10 mM HEPES, pH 7.9; 1.5 mM MgCl_2_; 10 mM KCl; 0.1% NP40; 1× protease inhibitor cocktail (Roche, 04 693 116 001); 50 mM sodium fluoride; 0.2 mM sodium orthovanadate; 0.05 mM MG132 (Sigma-Aldrich, M7449); 1 mM PMSF). After leaving the resuspended cells for 5 min on ice, an equal volume of lysis buffer B (10 mM HEPES, pH 7.9; 1.5 mM MgCl_2_; 10 mM KCl; 0.1% NP40; 1× protease inhibitor cocktail (Roche); 50 mM sodium fluoride; 0.2 mM sodium orthovanadate; 0.05 mM MG132 (Sigma-Aldrich, M7449); 1 mM PMSF) was added to leave another 5 min on ice. Cells were lysed by pipetting up and down followed by passing through a 27.5-gauge needle (insulin syringe) for 10–12 times on ice. This was followed by centrifugation at 15,000*g* for 15 min, and the supernatant was collected. For in vivo samples, embryonic cortex (dorsal telencephalon) was collected at E12.5 and E14.5 to proceed with protein extraction as above.

### Co-IP

IP was performed using 2 µg of anti-YY1 antibody (mouse anti-YY1; Santa Cruz Biotechnology, sc-7341) and control mouse IgG from in vivo (embryonic cortex) and in vitro (P19 cells) samples. Anti-YY1 antibody was incubated with Protein G Magnetic Dynabeads at 4 °C for 1–3 h in IP 150 KCl buffer (25 mM Tris, pH 7.9; 5 mM MgCl_2_; 10% glycerol; 150 mM KCl; 0.1% NP40; 0.3 mM DTT; 1× protease inhibitor cocktail (Roche, 04 693 116 001), 50 mM sodium fluoride; 0.2 mM sodium orthovanadate; 0.05 mM MG132 (Sigma-Aldrich, M7449); 1 mM PMSF). Then, 0.05% NP40 was added to the protein and centrifuged at 17,530*g* for 15 min. The supernatant was collected and added with 0.1 mg ml^−1^ ethidium bromide to incubate for 30 min, followed by centrifugation at 17,530*g* for 15 min. The supernatant was then pre-cleared with Protein G Dynabeads for 1 h by end-over-end rotation at 4 °C. After pre-clearing, protein was added to the Dynabeads, which were previously incubated with anti-YY1 antibody, followed by overnight rotation at 4 °C. The supernatant was removed after overnight incubation, followed by four washes using PBS with protease inhibitors (0.3 mM DTT; 1× protease inhibitor cocktail (Roche, 04 693 116 001); 50 mM sodium fluoride; 0.2 mM sodium orthovanadate; 0.05 mM MG132 (Sigma-Aldrich, M7449); 1 mM PMSF). The proteins bound to the beads were eluted using 2× Laemmli buffer, by heating at 95 °C for 5 min. Proteins were separated from beads using a magnet and proceeded to western blotting to visualize the immunoprecipitated proteins.

### Western blot

The immunoprecipitated proteins were run on 12% SDS-PAGE gels at 70 V during stacking and 120 V while resolving. The proteins were transferred to PVDF membranes (1620177, Bio-Rad) in transfer buffer (25 mM Tris; 192 mM glycine; 20% methanol, pH 8.3) at 40 V overnight at 4 °C after the SDS-PAGE. Membranes were blocked in TBST (10 mM Tris; 100 mM NaCl, pH 7.4; 0.1% Tween 20) with 5% (w/v) skim milk for 1 h at room temperature and then incubated with primary antibodies overnight at 4 °C. Membranes were washed 3 × 10 min in TBST and then incubated for 1 h at room temperature with 1/50,000 dilutions of horseradish peroxidase (HRP)-coupled secondary antibodies (anti-rabbit IgG, 7074S, Cell Signaling Technology). Membranes were washed 3 × 10 min at room temperature and then processed with ECL Plus Western Blotting Reagent (29018904, GE Healthcare) before developing with X-ray film (1141J52, LabForce) and a Bio-Rad ChemiDoc MP Imaging System. The primary antibodies used were rabbit anti-YY1 (Invitrogen, MA5-32052), rabbit anti-Neurogenin2 (Invitrogen, PA5-78556) and rabbit anti-Ezh2 (Cell Signaling Technology, 5246).

### Single-cell multiome pre-processing

Single-cell multiome reads were aligned to the *Mus musculus* reference genome (GRCm38, mm10), and the quantification was performed using cellranger-arc-2.0.1. Data were analyzed using Signac (version 1.7.0)^68^ and ArchR^44^. The quality control (QC) metrics are reported in Supplementary Table 1.

### Single-cell multiome QC

We eliminated low-information content cells based on the following selection criteria: cells where fewer than 1,000 genes and 1,000 unique molecular identifiers (UMIs) (from the gene expression library) and fewer than 8,000 unique fragments per cell, transcription start site (TSS) enrichment <1 and nucleosome signal <0.2 (from the ATAC library) were detected. To avoid including possible doublets in the further analysis, cells where more than 30,000 genes (from the gene expression library) and more than 125,000 unique fragments, TSS enrichment >20 and nucleosome signal >2 (from the ATAC library) were eliminated. Nucleosome signal and TSS enrichment were calculated using Signac (version 1.7.0)^68^ and plotted using ggplot2. Fragment lengths were calculated using ArchR^44^ and plotter using ggplot2. Upon filtering out the low-quality cells from all the conditions, the number of cells from the Astro condition was balanced with the other conditions.

### Single-cell multiome clustering and dimension reduction

The individual modalities (gene expression and ATAC) were normalized and processed using Signac^68^ and Seurat (version 4.0)^33^. In brief, peak calling was performed on pseudobulk aggregate per condition using MACS2. A high-quality union peak set was identified by merging the individual peaks and filtering out peaks, which overlapped with a list of blacklisted regions. The count matrix for the high-quality peak set was generated and incorporated into a Seurat object. It was subjected to TF-IDF normalization followed by SVD as described previously. For the gene expression modality, after log transformation, variance-stabilizing transformation was used to perform feature selection. Principal component analysis was performed using the first 20 dimensions. We then computed a joint neighbor graph that represents both gene expression and chromatin accessibility using FindMultiModalNeighbors. We then applied Louvain clustering to cluster cells (resolution = 0.2, n.start = 20, n.iter = 30, algorithm = 1), and the cell clusters were visualized using UMAP (min.dist = 0.5, spread = 1.5, n.components = 2 L). Cluster identity was determined based on the top 40 differentially expressed genes (MAST, minimum expression change of 0.25 and expressed by at least 25% of the cells in the cluster)^69^ as well as known marker genes.

### Single-cell multiome pseudotime analysis

Maturation pseudotime analysis was implemented on the QC-approved cells using Monocle3 (refs. ^34–37^). The UMAP coordinates was retained from Seurat and used to build the cds object in Monocle3. Cells in cluster iN_1 were selected as the root cells, and a trajectory graph was constructed using the following parameters: minimal_branch_len = 5, maxiter = 30. The change in gene expression along the constructed trajectory was calculated by fitting a generalized additive model employing cubic regression splines and REML smoothing. The resulting values were rescaled from 0 to 1.

### Single-cell multiome motif and ChIP-seq accessibility variation and TF footprinting

The calculation of motif accessibility deviation scores using position weight matrices obtained from the JASPAR2000 database and Ngn2 ChIP-seq was performed as described previously^19^ using the ChromVar implementation in Signac^68^. TF footprints were calculated using ArchR^44^ and visualized using ggplot2.

### Identification of putative EGPs

To link putative enhancers with their target genes, we used ArchR with empirical *P* value estimation and *k* = 50. We distinguish among positively correlated (*r* > 0.35; false discovery rate (FDR) < 0.1), negatively correlated (*r* < 0.35; FDR < 0.1) and non-correlated pairs (−0.35 < *r* < 0.35) that are separated by at least 5 kb but by no more than 500 kb.

### Identification of predicted gene targets based on ChIP-seq and EGPs

We reasoned that we can predict direct targets of a TF either by using available ChIP-seq peaks or based on the enrichment of the TF motif in the positively correlated EGPs. First, we identified all EGPs that contained the corresponding ChIP-seq peak or TF motif (either in the distal region or in the promoter region). Thereafter, we calculated the ‘gene linkage score’ by adding up the *r*^2^ from each pair per gene (if the peak/motif was contained in the promoter, we used a value of *r* = 1). To calculate enrichment, we used background ATAC peaks with similar GC content and determined significance using a hypergeometric test. A potential limitation of this method is that the significance of peak/motif enrichment for genes with very few identified pairs cannot be accurately calculated.

### Single-cell RNA-seq with 3′ cell multiplexing

The experimental conditions were labeled according to the manufacturer’s instructions with the following CellPlex reagents from the 3′ CellPlex Kit set A (10x Genomics, PN:1000261): Yy1 WT (CMO309), Yy1 KO (CMO310), Yy1 WT/Ngn2^+^ (CMO311) and Yy1 KO/Ngn2^+^ (CMO312). Approximately 25,000 events per condition were FACS sorted (Yy1 WT; untransduced, Yy1 KO; RFP^+^, Yy1 WT/Ngn2^+^; GFP^+^, Yy1 KO/Ngn2^+^; RFP^+^GFP^+^) into an Eppendorf tube. Approximately 33,000 cells were loaded onto a Chromium Next GEM ChIP G (10x Genomics, PN:2000177) to obtain a targeted cell recovery of 20,000 cells. The gene expression library (PN:3000431, single cell 3′ v3) and the cell multiplexing library (PN:3000482) were prepared according to the manufacturer’s protocol (CG000388, Rev A). The gene expression library and the cell multiplexing library were quality controlled using the Agilent 2100 Bioanalyzer, and the libraries were sequenced according to the manufacturer’s specifications.

### Single-cell RNA-seq pre-processing

Single-cell RNA-seq reads were aligned to the *Mus musculus* reference genome (GRCm38, mm10), and the sample assignment and quantification were performed using cell ranger multi in cellranger-6.0.0. The QC metrics are reported in Supplementary Table 1.

### Single-cell RNA-seq quality control

We eliminated low-information content cells based on the following selection criteria: cells where fewer than 1,000 genes and 2,500 UMIs were detected. To exclude dead cells, we filtered out cells containing more than 20% mitochondrial reads. To avoid including doublets in the further analysis, cells containing more than 6,000 genes were excluded.

### Single-cell RNA-seq clustering and dimension reduction

Seurat (version 4.0) was used to analyze the cells that passed the filtering steps. The data were normalized using SCTransform, and principal component analysis was performed using the first 25 dimensions. We applied Louvain clustering (resolution = 0.6, n.start = 20, n.iter = 20), and the data were visualized by UMAP projection (min.dist = 0.5, spread = 1.5, n.components = 2 L). Cluster identity was determined based on the top 40 differentially expressed genes (MAST, minimum expression change of 0.25 and expressed by at least 25% of the cells in the cluster)^69^.

### Bulk ATAC-seq analysis

The ATAC-seq FASTQ files were demultiplexed using Je (version 1.2)^70^, and the demultiplexed reads were aligned to the mouse genome (GRCm38, mm10). Post-alignment read filtering, peak calling and irreproducible discovery rate (IDR)-based peak filtering were performed by implementing the ENCODE ATAC-seq pipeline. The sequencing and QC metrics are listed in the form of a supplementary data table. The bigWig coverage track was generated using deepTools (version 3.1.3)^71^. The plotting of the ATAC-seq signal at genomic features was performed using SeqPlots^72^. The QC metrics are reported in Supplementary Table 1.

### Bulk RNA-seq analysis

The RNA-seq FASTQ files were demultiplexed using Je (version 1.2)^70^; demultiplexed reads were aligned to the mouse genome (GRCm 38, mm10) using STAR (version 2.7.1a)^73^; and read counts per gene were obtained by using the –quantMode GeneCounts option. Further analysis was performed using DEseq2 (ref. ^74^) in RStudio. The result table for pairwise comparison between PmutNgn2 versus Ngn2 was used the input to generate the GO term enrichment bubble plot in the R package clusterProfiler^75^. The QC metrics are reported in Supplementary Table 1.

### ChIP-seq analysis

The ChIP-seq FASTQ files were demultiplexed using Je (version 1.2)^70^; demultiplexed reads were aligned to the mouse genome (GRCm38, mm10); and post-alignment read filtering, peak calling and IDR-based peak filtering were performed by implementing the ENCODE ChIP-seq pipeline. The bigWig coverage track was generated using deepTools (version 3.1.3)^71^. The plotting of the ChIP-seq signal at genomic features was performed using the R package SeqPlots^72^. The QC metrics are reported in Supplementary Table 1.

### CUT&RUN analysis

CUT&RUN data were uniformly processed using CUT&RUN tools 2.0 (ref. ^76^). Peaks were called using MACS2, and the bigWig coverage track was generated using deepTools (version 3.1.3)^71^. The QC metrics are reported in Supplementary Table 1.

### Hi-C mapping and QC

FASTQ files from the Methyl-HiC were mapped to the mouse genome (GRCm38, mm10) by employing JuiceMe^77^. Further analysis was performed only with uniquely mapping reads (mapq score > 30). After the elimination of polymerase chain reaction (PCR) duplicates, the translation of reads into a pair of fragment ends (fends) was achieved by the association of each read with its downstream fend. MethylDackel was used to assess CpG methylation, which entailed the elimination of the initial six nucleotides in the ‘mergeContext’ mode. Pooling of reads from individual replicates was performed, and, for a cytosine to be considered for further analysis, it had to be in the CpG context and possess at least 10× total coverage. In case of Hi-C, exclusion of reads was based on the following criteria: mapped to the same restriction fragment and separated by less than 1 kb. The QC metrics are reported in Supplementary Table 1.

### Hi-C data processing

Filtered fend-transformed read pairs were imported into the following genome database: mm10 after conversion into ‘misha’ tracks. The Shaman package was used for read pair normalization (https://tanaylab.bitbucket.io/shaman/index.html), and the calculation of the Hi-C score was performed by employing *k*-nearest neighbors (kNN)^22^.

### Contact probability, insulation and TAD boundary calling

The calculation of contact probabilities as a function of genomic distance was previously described^22^. The insulation score, which is used to define insulation on the basis of observed contacts, was also previously described ^19,22,78^, and differential TAD boundaries were identified using insulation score^19,22^.

### Compartments and compartment strength

The calculation of contact matrices dominant eigenvector, which have been binned at 250 kb, was previously described and performed using publicly available scripts (https://github.com/dekkerlab/cworld-dekker)^79^. Compartment strength was determined by plotting the log_2_ ratio of observed versus expected contacts (intrachromosomal separated by at least 10 Mb) between A–A, B–B or A–B domains. A ratio between the sum of observed contacts within the A and B compartments and the sum of intercompartment contacts was calculated to determine the compartment strength^19^.

### Average TAD contact enrichment

The calculation of insulation and contact enrichment within TADs was previously described^19,22^.

### Aggregated and individual contact strength at pairs of genomic features

Two complementary approaches were employed for the calculation of contact enrichment ratio at genomic feature pairs, such as Ngn2 ChIP-seq peaks or EGPs. Aggregated Hi-C maps were used to calculate the log_2_ ratio of observed versus expected contacts within a specified window size, which was centered on the feature of interest. The average enrichment ratio was also calculated for the following: contact strength in the center of the window versus each of the corners. Furthermore, the extraction of kNN-based Hi-C score for each pair in a 10-kb window centered around it and its representation as a scatter plot or box plots enabled the identification of pair-specific trends. Significance testing was performed by using the Wilcoxon rank-sum test.

### Reporting summary

Further information on research design is available in the Nature Portfolio Reporting Summary linked to this article.

## Online content

Any methods, additional references, Nature Portfolio reporting summaries, source data, extended data, supplementary information, acknowledgements, peer review information; details of author contributions and competing interests; and statements of data and code availability are available at 10.1038/s41593-024-01677-5.

### Supplementary information

Reporting Summary

Supplementary Tables Supplementary Table 1: QC metrics for genomic datasets. Table 2: List of genes and their associated enhancers that display coordinated changes in 3D genome remodeling and DNA methylation (related to Figs. 4a and 5i,k). Table 3: List of the differentially expressed genes from each.experimental condition in the single-cell RNA-seq datasets generated to study the effect of *Yy1* deletion on direct neuronal reprogramming (related to Fig. 6j). Statistical sigficance was identified using MAST. Table 4: List of antibodies used in the study.

Supplementary Video 1 Time-lapse imaging of Ngn2-mediated direct neuronal reprogramming of mouse astrocytes to neurons. Scale bar, 50 μm.

Supplementary Video 2 Time-lapse imaging of PmutNgn2-mediated direct neuronal.reprogramming of mouse astrocytes to neurons. Scale bar, 50 μm.

### Source data

Source Data Fig. 1 Statistical source data.

Source Data Fig. 7 Statistical source data.

Source Data Fig. 8 Unprocessed western blots.

## Data Availability

Sequencing data that support the findings of this study have been deposited in the Gene Expression Omnibus under accession code GSE208742. Previously published data that were re-analyzed in this study are available under the following accession codes: Methyl-HiC (GSE155677), Ngn2 ChIP-seq from mouse embryoid bodies (GSE114176) and Ngn2 ChIP-seq from mouse embryonic cortex (GSE63621). Source data are provided with this paper.

## Code availability

The code used for generating the data and all the figures is freely available at https://github.com/BonevLab/Pereira_et_al_NatNeuro2024. The R package to compute the expected tracks and the Hi-C scores is available at https://github.com/tanaylab/shaman.
