# Supplementary material for: Direct neuronal reprogramming of mouse astrocytes is associated with multiscale epigenome remodeling and requires Yy1
Source: Nat Neurosci. 2024 Jul 2;27(7):1260–73. doi: 10.1038/s41593-024-01677-5 (PMC11239498; doi:10.1038/s41593-024-01677-5)
Supplement: Supplementary file 1 — Reporting Summary [file 41593_2024_1677_MOESM1_ESM.pdf]

nature portfolio

NN-A80379B
Corresponding author(s): Boyan Bonev and Magdalena Goetz

Last updated by author(s): Apr 22, 2024

Reporting Summary

Nature Portfolio wishes to improve the reproducibility of the work that we publish. This form provides structure for consistency and transparency in reporting. For further information on Nature Portfolio policies, see our Editorial Policies and the Editorial Policy Checklist.

Statistics

For all statistical analyses, confirm that the following items are present in the figure legend, table legend, main text, or Methods section.

n/a	Confirmed	
☐	☒	The exact sample size (*n*) for each experimental group/condition, given as a discrete number and unit of measurement
☐	☒	A statement on whether measurements were taken from distinct samples or whether the same sample was measured repeatedly
☐	☒	The statistical test(s) used AND whether they are one- or two-sided<br>*Only common tests should be described solely by name; describe more complex techniques in the Methods section.*
☐	☒	A description of all covariates tested
☐	☒	A description of any assumptions or corrections, such as tests of normality and adjustment for multiple comparisons
☐	☒	A full description of the statistical parameters including central tendency (e.g. means) or other basic estimates (e.g. regression coefficient) AND variation (e.g. standard deviation) or associated estimates of uncertainty (e.g. confidence intervals)
☐	☒	For null hypothesis testing, the test statistic (e.g. *F*, *t*, *r*) with confidence intervals, effect sizes, degrees of freedom and *P* value noted<br>*Give P values as exact values whenever suitable.*
☒	☐	For Bayesian analysis, information on the choice of priors and Markov chain Monte Carlo settings
☒	☐	For hierarchical and complex designs, identification of the appropriate level for tests and full reporting of outcomes
☐	☒	Estimates of effect sizes (e.g. Cohen's *d*, Pearson's *r*), indicating how they were calculated

Our web collection on statistics for biologists contains articles on many of the points above.

Software and code

Policy information about availability of computer code

Data collection	For live-imaging microscopy, a Zeiss Cell Observer (Zeiss) was employed to perform continuous live imaging of astrocyte-to-neuron conversion. The acquisition of images was performed as follows: phase contrast images and fluorescent images (GFP) were captured every 20 minutes and 4 hours respectively, with a 10x phase contrast objective (Zeiss) and an AxioCam HRm camera. The Zeiss AxioVision 4.7 software was controlled by a custom-made VBA module (TAT, Prof. Timm Schroeder, ETH Zürich, Switzerland). The acquisition of microscopy images was performed using an AxioM2 epifluorescence microscope (Carl Zeiss) or LSM710 laser-scanning confocal (Carl Zeiss) and Zen2 software (Version 2.0.0.0, Carl Zeiss)
Data analysis	ImageJ (1.53q), FlowJo (10.8.1), Cellranger-arc-2.0.1, Cellranger-6.0.0, pheatmap_1.0.12, TxDb.Mmusculus.UCSC.mm10.knownGene_3.10.0, TFBSTools_1.32.0, monaLisa_1.3.1, BSgenome.Mmusculus.UCSC.mm10_1.4.3, org.Mm.eg.db_3.14.0, SummarizedExperiment_1.24.0, GenomicRanges_1.46.1, IRanges_2.28.0, BiocGenerics_0.40.0, matrixStats_0.61.0, cowplot_1.1.1, ggplot2_3.3.5, Seurat_4.1.0, SeuratObject_4.0.4, misha_4.1.0, magrittr_2.0.3, GenomicFeatures_1.46.5, RColorBrewer_1.1-3, motifmatchr_1.16.0, JASPAR2020_0.99.10, tglkmeans_0.3.3, BSgenome_1.62.0, Biostrings_2.62.0, MAST, clusterProfiler_4.2.2, ArchR, Signac_1.7.0, AnnotationDbi_1.56.2, GenomeInfoDb_1.30.1, dplyr_1.0.8, Je (v1.2), Deeptools (v3.1.3), Seqplots, STAR (v2.7.1a), ENCODE ATAC-seq pipeline, ENCODE ChIP-seq pipeline. The code used for generating the data and all the figures are freely available under https://github.com/BonevLab/Pereira_et_al_NatNeuro2024. The R package to compute the expected tracks and the Hi-C scores is available at https://github.com/tanaylab/shaman.

For manuscripts utilizing custom algorithms or software that are central to the research but not yet described in published literature, software must be made available to editors and reviewers. We strongly encourage code deposition in a community repository (e.g. GitHub). See the Nature Portfolio guidelines for submitting code & software for further information.

Data

Policy information about availability of data

All manuscripts must include a data availability statement. This statement should provide the following information, where applicable:

- Accession codes, unique identifiers, or web links for publicly available datasets
- A description of any restrictions on data availability
- For clinical datasets or third party data, please ensure that the statement adheres to our policy

All raw and processed sequencing data are available in the Gene Expression Omnibus (GEO) repository: GSE208742. Previously published data that were re-analysed in this study are available under the following accession codes: Methy-HiC (GSE155677), Ngn2 ChIP-seq from mouse embryoid bodies (GSE114176), Ngn2 ChIP-seq from mouse embryonic cortex (GSE63621)

Research involving human participants, their data, or biological material

Policy information about studies with human participants or human data. See also policy information about sex, gender (identity/presentation), and sexual orientation and race, ethnicity and racism.

Reporting on sex and gender	N/A
Reporting on race, ethnicity, or other socially relevant groupings	N/A
Population characteristics	N/A
Recruitment	N/A
Ethics oversight	N/A

Note that full information on the approval of the study protocol must also be provided in the manuscript.

Field-specific reporting

Please select the one below that is the best fit for your research. If you are not sure, read the appropriate sections before making your selection.

☒ Life sciences ☐ Behavioural & social sciences ☐ Ecological, evolutionary & environmental sciences

For a reference copy of the document with all sections, see nature.com/documents/nr-reporting-summary-flat.pdf

Life sciences study design

All studies must disclose on these points even when the disclosure is negative.

Sample size	No statistical methods were used to pre-determine the sample size. Sample sizes for all genomic datasets are provided in the Supplementary data table 1. Sample sizes for scRNA-seq/scATAC-seq were chosen based upon the ability to get an unbiased view on the cellular composition for each sample with affordable cost. Sample size for Methyl-HiC were chosen in order to obtain representative data, ensure replication of results and based on analogous studies in the field. Sample sizes for ChIP-seq, bulk ATAC-seq and bulk RNA-seq were chosen in accordance with ENCODE experiment guidelines. Sample sizes for CUT&RUN, immunocytochemistry, live-imaging and co-immunoprecipitation were chosen based on analogous studies in the field. All the analogous studies have been cited in the manuscript
Data exclusions	No samples were excluded from the analysis. In the case of scRNA-seq and scATAC-seq data, cells with low quality control values were excluded on the basis of criteria described in the Methods section.
Replication	The experimental findings were validated with functional studies (conditional knockouts of the candidate gene, Yy1) and molecular analysis (scRNA-seq and bulk ATAC-seq). All attempts of replication were successful. In case of the multiome experiment (scRNA + scATAC-seq), the data is from N = 1, based upon the ability to get an unbiased view on the cellular composition for each sample with affordable cost. Methy-HiC and co-immunoprecipitation was performed in biological duplicates. ChIP-seq, bulk RNA-seq, immunocytochemistry and live-imaging experiments were performed in biological triplicates. Bulk ATAC-seq was performed in 2-3 biological replicates. CUT&RUN was performed in 1-3 biological replicates. The biological replicates were performed independently and all attempts of replication were successful
Randomization	The primary astrocytes, transduced with either the GFP, Ngn2 or PmutNgn2 lentivirus, were obtained from the same litter of mice. In case of the primary astrocytes obtained from the Yy1tm2Yshi line for the functional studies (conditional knockouts of the candidate gene, Yy1), the wild-type, heterozygote and homozygote genotypes were obtained from same litter of mice by crossing two heterozygote mice.
Blinding	All the data analysis for immunocytochemistry and live-imaging was performed by the investigators in a blinded manner. The genomic experiments and the associated data analysis were not blinded since they did not involve subjective measurements.

Reporting for specific materials, systems and methods

We require information from authors about some types of materials, experimental systems and methods used in many studies. Here, indicate whether each material, system or method listed is relevant to your study. If you are not sure if a list item applies to your research, read the appropriate section before selecting a response.

Materials & experimental systems

n/a	Involved in the study
☐	☒ Antibodies
☒	☐ Eukaryotic cell lines
☒	☐ Palaeontology and archaeology
☐	☒ Animals and other organisms
☒	☐ Clinical data
☒	☐ Dual use research of concern
☒	☐ Plants

Methods

n/a	Involved in the study
☐	☒ ChIP-seq
☐	☒ Flow cytometry
☒	☐ MRI-based neuroimaging

Antibodies

Antibodies used

The primary antibodies used in this study are listed below:
1) Anti-beta-III-tubulin (1:200 Mouse IgG2b Sigma-Aldrich Cat #T8660)
2) Anti-Gfap (1:500 Mouse IgG1 Sigma-Aldrich Cat# G3893)
3) Anti-Dcx (1:2000 Guinea pig Merck/Millipore AB2253)
4) Anti-NeuN (1:300 Mouse IgG1 Merck/Millipore MAB377)
5) Anti-GFP (1:300 Chicken Aves Lab GFP-1020)
6) Anti-RFP (1:1000 Rabbit Rockland/Biomol 600-401-379)
7) Anti-FLAG M2 (1:50 Mouse Sigma F1804)
8) DYKDDDDK Tag (D6W5B) (1:50 Rabbit mAB Anti-FLAG M2 antibody Cell Signaling Technology #14793)
9) Anti-Yy1 (D5D9Z) (1: 10 Rabbit mAb Cell Signaling Technology #46395)
10) Anti-FLAG (1:25 Mouse mAb Sigma #F3165)
11) Anti-Rad21 (1: 10 Rabbit pAb Biozol #GTX106012)
12) Anti-H3K27Ac (1:50 Rabbit pAb Active Motif #39133)
13) Anti-Yy1 (H-10) (1:125 Mouse mAb Santa Cruz #sc-7341)
14) Control mouse IgG (1:125 Mouse isotype control Invitrogen Cat# 02-6502)

The secondary antibodies used in this study are listed below:
1) Anti-chicken Alexa Fluor 488 (1:500 Donkey Jackson Immuno 703-545-155)
2) Anti-guinea pig Cy3 (1:500 Donkey Dianova 706-166-148)
3) Anti-rabbit Cy3 (1:500 Donkey Dianova 711-165-152)
4) Anti-mouse IgG1 Alexa Fluor 647 (1:500 Goat ThermoFisher A21240)
5) Anti-mouse IgG2b Alexa Fluor 647 (1:500 Goat ThermoFisher A21242)

Validation

The antibodies were validated by the corresponding manufacturer:
Anti-beta-III-tubulin (https://www.sigmaaldrich.com/FR/fr/product/sigma/t8660)
Anti-Gfap (https://www.sigmaaldrich.com/FR/fr/product/sigma/g3893)
Anti-Dcx (https://www.merckmillipore.com/FR/fr/product/Anti-Doublecortin-Antibody,MM_NF-AB2253?ReferrerURL=https%3A%2F%2Fwww.google.com%2F)
Anti-NeuN (https://www.merckmillipore.com/FR/fr/product/Anti-NeuN-Antibody-clone-A60,MM_NF-MAB377?ReferrerURL=https%3A%2F%2Fwww.google.com%2F)
Anti-GFP (https://www.aveslabs.com/products/anti-green-fluorescent-protein-antibody-gfp)
Anti-RFP (https://www.biomol.com/products/antibodies/primary-antibodies/epitope-tag/anti-red-fluorescent-proteinrfp-600-401-379)
Anti-chicken Alexa Fluor 488 (https://www.jacksonimmuno.com/catalog/products/703-545-155)
Anti-guinea pig Cy3 (https://www.dianova.com/en/shop/706-166-148-donkey-fab2-anti-guinea-pig-igg-hl-cy3-minxbockgohshohumsrbrtsh/)
Anti-rabbit Cy3 (https://www.dianova.com/en/shop/711-165-152-donkey-igg-anti-rabbit-igg-hl-cy3-minx-bockgogphshohumsrtsh/)
Anti-mouse IgG1 Alexa Fluor 647 (https://www.thermofisher.com/antibody/product/Goat-anti-Mouse-IgG1-Cross-Adsorbed-Secondary-Antibody-Polyclonal/A-21240)
Anti-mouse IgG2b Alexa Fluor 647 (https://www.thermofisher.com/antibody/product/Goat-anti-Mouse-IgG2b-Cross-Adsorbed-Secondary-Antibody-Polyclonal/A-21242)
Anti-FLAG M2 (https://www.sigmaaldrich.com/FR/fr/product/sigma/f1804?gclid=CjwKCAjwyaWZBhBGEiwACslQo0hVpX6fD7j2FFW24k3hnxTCSz9I3iqbLytKfIGwH9lcLbvi6pczVhoCQqcQAvD_BwE&gclsrc=aw.ds)
DYKDDDDK Tag (D6W5B) Anti-FLAG M2 antibody (https://www.cellsignal.com/products/primary-antibodies/dykddddk-tag-d6w5brabbit-
mab-binds-to-same-epitope-as-sigma-aldrich-anti-flag-m2-antibody/14793)
Anti-Yy1 (https://www.cellsignal.com/products/primary-antibodies/yy1-d5d9z-rabbit-mab/46395)
Anti-FLAG (https://www.sigmaaldrich.com/FR/fr/product/sigma/f3165)
Anti-Rad21 (https://www.biozol.de/de/product/gtx106012)
Anti-H3K27Ac (https://www.activemotif.com/catalog/details/39133/histone-h3-acetyl-lys27-antibody-pab)

Anti-YY1 (https://www.scbt.com/p/yy1-antibody-h-10)
Control Mouse IgG (https://www.thermofisher.com/antibody/product/Mouse-IgG-Isotype-Control/02-6502)

Animals and other research organisms

Policy information about studies involving animals; ARRIVE guidelines recommended for reporting animal research, and Sex and Gender in Research

Laboratory animals	M2rtTA (#006965) and Yy1tm2Yshi mice (#014649) were obtained from Jackson Laboratory. Mice were housed in individually ventilated cage systems in a room with a room temperature of 22 +/- 2 °C and 55 +/- 10 % relative humidity. The age of breeding pairs for the M2rtTA (#006965) and Yy1tm2Yshi (#014649) mice lines was between 2 - 6 months. The age of the mice used in all experiments was postnatal day 5-6
Wild animals	No wild animals were used in this study
Reporting on sex	The sex of the animals was not considered in the study design
Field-collected samples	No field collected samples were used in this study
Ethics oversight	All experimental procedures for this study were performed at the Biomedical Center, LMU Munich, in accordance with German and European Union guidelines and were approved by the government of Upper Bavaria.

Note that full information on the approval of the study protocol must also be provided in the manuscript.

Plants

Seed stocks	N/A
Novel plant genotypes	N/A
Authentication	N/A

ChIP-seq

Data deposition

☒ Confirm that both raw and final processed data have been deposited in a public database such as GEO.

☒ Confirm that you have deposited or provided access to graph files (e.g. BED files) for the called peaks.

Data access links<br>*May remain private before publication.*	All raw and processed sequencing data are available in the Gene Expression Omnibus (GEO) repository: GSE208742.
Files in database submission	The files submitted to the GEO repository include processed data files and raw fastq files. The processed data files are: Ngn2_chip_D2.bw and PmutNgn2_chip_D2.bw (merged bigwig file generated using Deeptools (v3.1.3) and Ngn2_ChIP_peaks.bed.gz and PmutNgn2_ChIP_peaks.bed.gz (compressed bed files of the high confidence set of Ngn2 ChIP peaks derived from the IDR analysis of biological replicates (ENCODE Transcription factor ChIP-seq pipeline)<br>The raw fastq files are:<br>1) Ngn2_ChIP_rep1_R1.fastq.gz<br>2) Ngn2_ChIP_rep2_R1.fastq.gz<br>3) Ngn2_ChIP_rep3_R1.fastq.gz<br>4) Ngn2_Input_rep1_R1.fastq.gz<br>5) Ngn2_Input_rep2_R1.fastq.gz<br>6) Ngn2_Input_rep3_R1.fastq.gz<br>7) PmutNgn2_ChIP_rep1_R1.fastq.gz<br>8) PmutNgn2_ChIP_rep2_R1.fastq.gz<br>9) PmutNgn2_ChIP_rep3_R1.fastq.gz<br>10) PmutNgn2_Input_rep1_R1.fastq.gz<br>11) PmutNgn2_Input_rep2_R1.fastq.gz<br>12) PmutNgn2_Input_rep3_R1.fastq.gz
Genome browser session<br>(e.g. UCSC)	A genome brower session has not been provided. We have provided a bigwig coverage file and bed file, which are available in the GEO database.

Methodology

Replicates	We performed and sequenced N= 3 biological replicates for the Ngn2 and PmutNgn2 ChIP-seq experiment
Sequencing depth	All the sequencing related metrics for the Ngn2 and PmutNgn2 ChIP-seq experiment have been described in Supplementary table 1 (file name: Supplementary_table_1.xlsx)
Antibodies	Anti-FLAG Mouse Sigma F1804. The antibody was validated by the manufacturer: https://www.sigmaaldrich.com/FR/fr/product/sigma/f1804?gclid=CjwKCAjwyaWZBhBGEiwACslQo0hVpX6fD7j2FFW24k3hnxTCSz9I3iqbLytKfIGwH9lcLbvi6pczVhoCQqcQAvD_BwE&gclsrc=aw.ds DYKDDDDK Tag (D6W5B) Rabbit mAB (Anti-FLAG M2 antibody) Cell Signaling Technology #14793. Antibody was validated by the manufacturer (https://www.cellsignal.com/products/primary-antibodies/dykddddk-tag-d6w5b-rabbit-mab-binds-to-same-epitopeas-sigma-aldrich-anti-flag-m2-antibody/14793)
Peak calling parameters	The ENCODE Transcription factor ChIP-seq pipeline was used for data processing and default peak calling parameters as specified by ENCODE were employed
Data quality	The ENCODE Transcription factor ChIP-seq pipeline was used for data processing and the data quality standards as specified by ENCODE were adhered to.
Software	ENCODE Transcription factor ChIP-seq pipeline

Flow Cytometry

Plots

Confirm that:

☒ The axis labels state the marker and fluorochrome used (e.g. CD4-FITC).

☒ The axis scales are clearly visible. Include numbers along axes only for bottom left plot of group (a 'group' is an analysis of identical markers).

☒ All plots are contour plots with outliers or pseudocolor plots.

☒ A numerical value for number of cells or percentage (with statistics) is provided.

Methodology

Sample preparation	Cells were prepared for FACS by washing them once with 1x PBS followed by trypsinization (0.05% trypsin in EDTA) for 5 minutes. The trypsinization reaction was stopped by adding astrocyte medium. The harvested cells were then washed twice with ice-cold PBS and centrifuged at 300g for 3 minutes at 4 degrees Celcius. The cells were resuspended in DMEM/F-12 (1:1) and a single-cell suspension was generated using a 40-um cell strainer. For Methly-HiC, astrocytes were stained for DAPI and only cells in G0 and G1 (single DNA content) as follows: Upon fixing with 1% formaldehyde and permeabilizing the cells as previously described, they were stained with DAPI (1:1000 dilution in wash buffer containing 1% BSA, 0.1% RNasin plus RNase inhibitor (Promega) in PBS). The cells were once with the wash buffer and subsequently, resuspended in PBS with 1% BSA and 1% RNasin plus RNase inhibitor, filtered through a 40-uM cell strainer
Instrument	FACS was performed by employing a BD FACSAria Fusion (BD Bioscience) using a 100-um nozzle.
Software	FlowJo (10.8.1)
Cell population abundance	The abundance of the sorted cells has been indicated in Extended data Fig. 5b
Gating strategy	After selecting singlets using forward and side scatter, cells in GOG1 were identified by genomic content based on DAPI staining. Thereafter, the cells were gated for GFP expression. The gating strategy has been depicted in Extended data Fig.5B.

☒ Tick this box to confirm that a figure exemplifying the gating strategy is provided in the Supplementary Information.
