# Supplementary material for: Direct neuronal reprogramming of mouse astrocytes is associated with multiscale epigenome remodeling and requires Yy1
Source: Nat Neurosci. 2024 Jul 2;27(7):1260–73. doi: 10.1038/s41593-024-01677-5 (PMC11239498; doi:10.1038/s41593-024-01677-5)

Figure 8i

Repeat 1

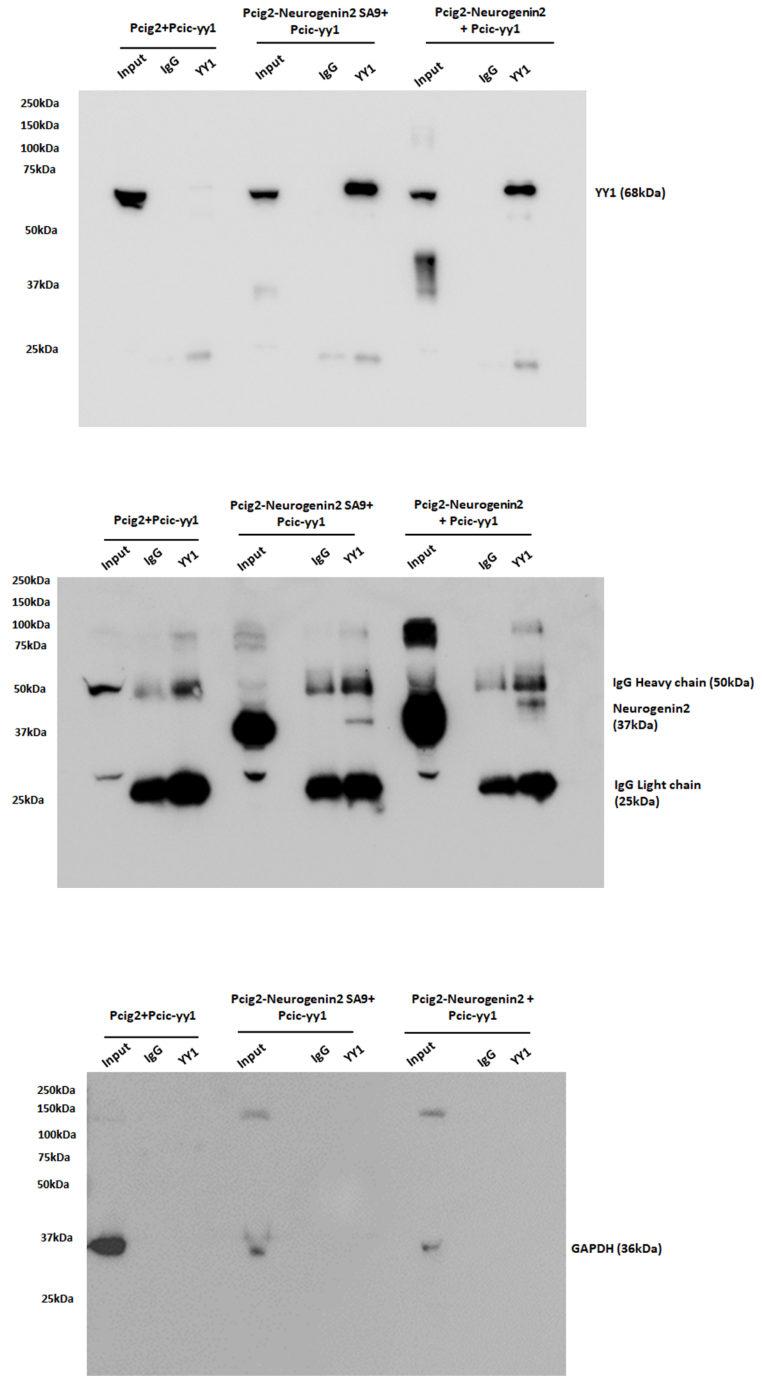

Repeat 2

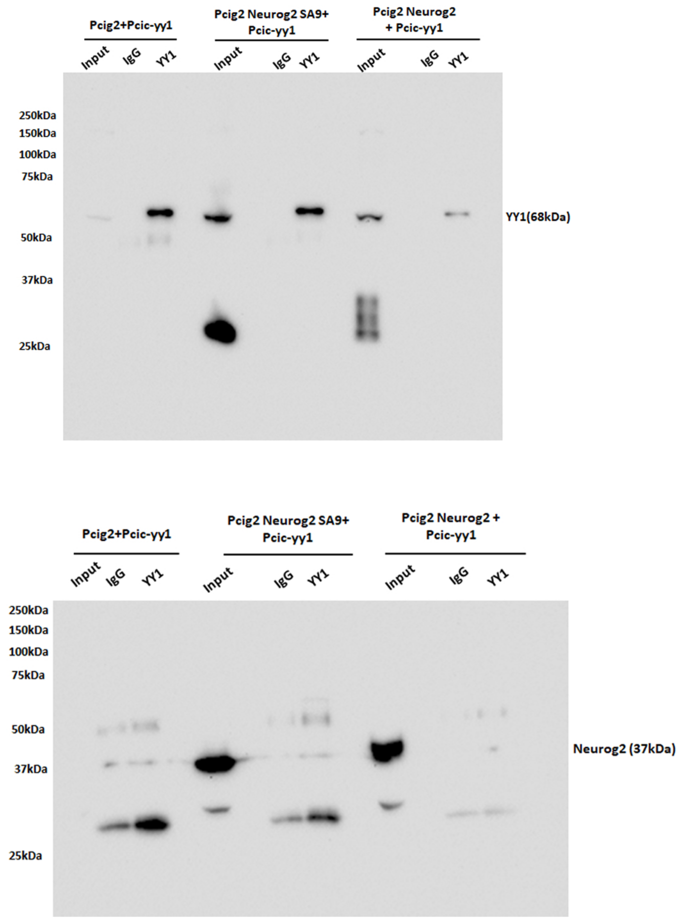

Figure 8j

Repeat 1 | **Repeat 2**

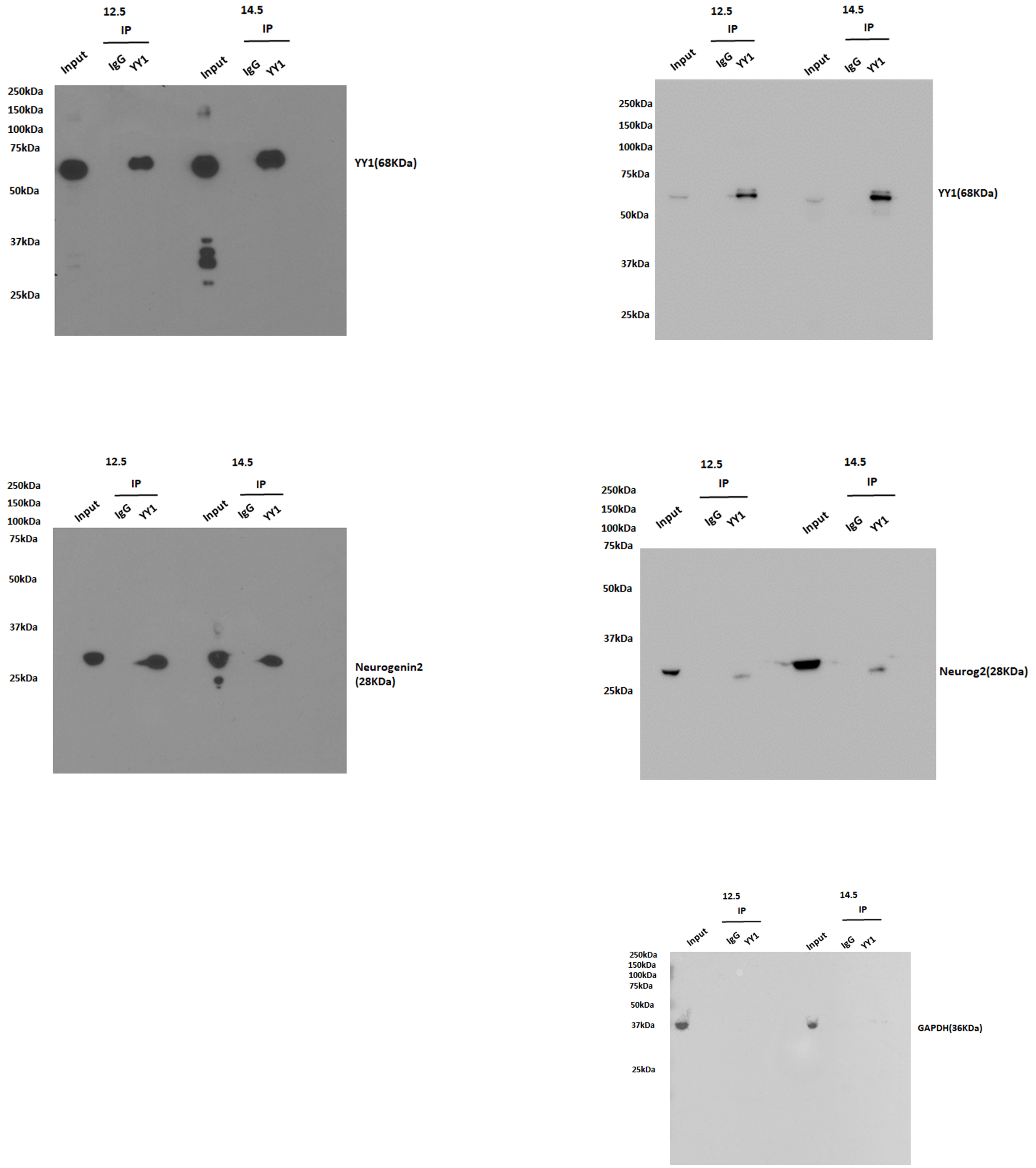

Supplement: Supplementary file 7 — Unprocessed western blots. [file 41593_2024_1677_MOESM7_ESM.pdf]
